# Galectin functions in cancer-associated inflammation and thrombosis

**DOI:** 10.3389/fcvm.2023.1052959

**Published:** 2023-02-17

**Authors:** Linus Kruk, Attila Braun, Erika Cosset, Thomas Gudermann, Elmina Mammadova-Bach

**Affiliations:** ^1^Walther-Straub-Institute for Pharmacology and Toxicology, Ludwig-Maximilians-University, Munich, Germany; ^2^Division of Nephrology, Department of Medicine IV, Ludwig-Maximilians-University Hospital, Munich, Germany; ^3^CRCL, UMR INSERM 1052, CNRS 5286, Centre Léon Bérard, Lyon, France; ^4^German Center for Lung Research (DZL), Munich, Germany

**Keywords:** galectins, cancer, inflammation, thrombosis, anti-cancer therapies

## Abstract

Galectins are carbohydrate-binding proteins that regulate many cellular functions including proliferation, adhesion, migration, and phagocytosis. Increasing experimental and clinical evidence indicates that galectins influence many steps of cancer development by inducing the recruitment of immune cells to the inflammatory sites and modulating the effector function of neutrophils, monocytes, and lymphocytes. Recent studies described that different isoforms of galectins can induce platelet adhesion, aggregation, and granule release through the interaction with platelet-specific glycoproteins and integrins. Patients with cancer and/or deep-venous thrombosis have increased levels of galectins in the vasculature, suggesting that these proteins could be important contributors to cancer-associated inflammation and thrombosis. In this review, we summarize the pathological role of galectins in inflammatory and thrombotic events, influencing tumor progression and metastasis. We also discuss the potential of anti-cancer therapies targeting galectins in the pathological context of cancer-associated inflammation and thrombosis.

## 1. Introduction

Galectins are β-galactoside-binding proteins, belonging to the protein family of lectins that share a common amino acid sequence and the carbohydrate recognition domain (CRD) (1). They are expressed in vertebrates, invertebrates, and unicellular eukaryotic organisms (protists), indicating their fundamental functions during evolution (2). Although galectins are distributed in many tissues, some of the isoforms are more specifically expressed. Galectins regulate the cell cycle, inflammation, immune response, cell adhesion, and cell signaling (1, 3). Galectins are also important players in cancer progression and metastasis by mediating interactions between tumor and tumor microenvironment (1, 3). Galectins are synthesized on free ribosomes with a frequently acetylated carboxy-terminal end (4). Galectins are secreted by an unconventional transport pathway since no signal peptide is detected in these proteins (5). Most studies focused on the extracellular effects of galectins; they can bind plasma membrane proteins and interact with extracellular matrix (ECM) components (6). However, galectins have also important intracellular functions, described in the cytoplasm, nucleus, mitochondria, exosomes, and lysosomes (5, 7). Furthermore, galectins are involved in intracellular trafficking by participating in the apical transport; stabilizing, and sorting of glycoproteins toward their destination (7). Some of the galectin isoforms play a pivotal role in the biogenesis of endocytic vesicles (8). Although most of the extracellular ligands of galectins are glycosylated, the main parts of intracellular ligands are not glycosylated. The gene expression of galectins is regulated in a tissue-specific and developmental stage-dependent manner, including many factors, divalent or multivalent protein complexes, and specific counter-receptors (9). Cancer is one of the pathological conditions that dysregulate gene expression of galectins and these alterations may contribute to common hallmarks of cancers such as neoplastic transformation, resistance to apoptosis, angiogenesis, or tumor metastasis (7). Current research showed that galectins can modulate immune responses and inflammatory effects on tumor cells and may help cancer cells to escape immune surveillance and form metastases (1). Furthermore, galectins bind platelet receptors, and are involved in platelet adhesion and activation, inducing pro-thrombotic events, as observed during atherosclerosis and venous thromboembolism (10–12). In this review, we focus on multiple functions of galectins, which modulate not only tumor cell-autonomous functions but also inflammation and thrombosis, thereby increasing cancer malignancy.

## 2. Structure of galectins

**Figure 1:**
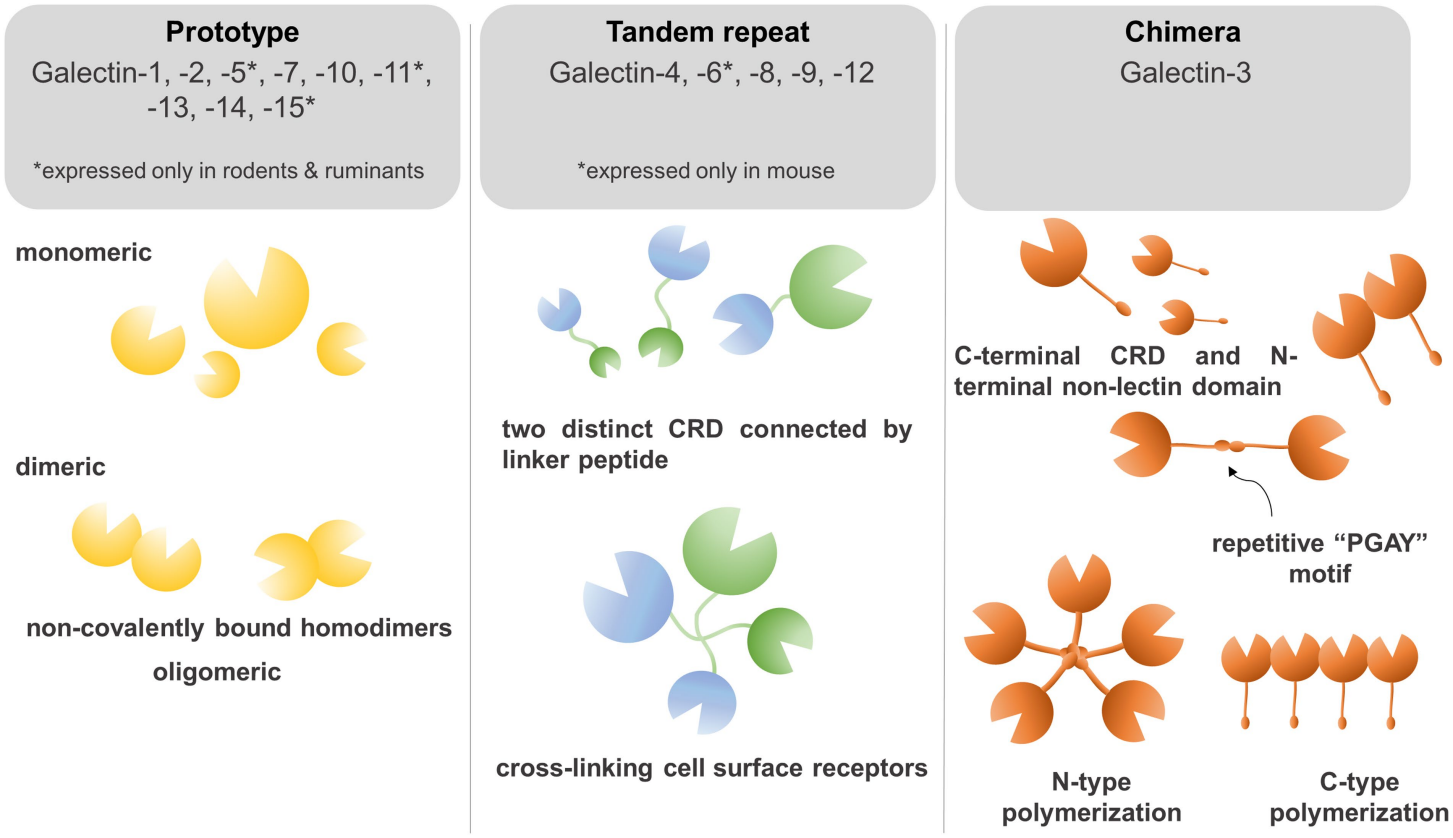
Galectins bind to β-galactose-containing glycoconjugates through a carbohydrate recognition domain (CRD). Based on their CRD domain structures, mammalian galectins were classified into proto, chimera, and tandem-repeat isoforms. Prototype galectins (Galectin −1, −2, −5, −7, −10, −11, −13, −14, and −15) contain an individual CRD domain per polypeptide and form non-covalently linked homodimers. Tandem-repeat galectins (Galectin −4,− 6, −8, −9, and −12) are composed of two CRDs connected by an unstructured linker peptide that allows for crosslinking of galectins on the cell surface. The chimera-type galectin-3 consists of one CRD domain on the C-terminus and a non-lectin N-terminus which represents two phosphorylation sites crucial for subcellular trans-localization. The N-terminus repetitive “PGAY” motif allows for N-type polymerization to pentamers, whereas multiple CRD domains can form β-sandwich structures.

To determine the structure of galectins it is necessary to understand the biochemical properties of galectins which crosslink glycoconjugates on different receptors thereby modulating diverse signaling pathways. Galectins are carbohydrate-binding proteins, characterized by the ability to bind β-galactose-containing glycoconjugates, which is maintained through a carbohydrate recognition domain (CRD) (13, 14). Based on their CRD domain structures, mammalian galectins were classified into proto, chimera, and tandem-repeat isoforms (Figure 1). Prototype of galectins (Galectin −1, −2, −5, −7, −10, −11, −13, −14, and −15) contains one CRD domain per polypeptide and non-covalently linked homodimers (9, 14). Tandem-repeat galectins (Galectin-4,- 6, −8, −9, and −12) are composed of two CRDs that are connected by an unstructured linker peptide (9, 14). Galectin-5 and galectin-6 are expressed in rodents, but not in humans, whereas galectin-11 is found in sheep and galectin-15 was detected in sheep and goats (13, 15). Galectin-3 is a unique member of chimeric isoforms in mammalians, consisting of one CRD domain on the C-terminus, fused to a non-lectin N-terminus which represents two phosphorylation sites (Ser6, Ser12). The phosphorylation status of galectin-3 regulates subcellular localization and translocation of the protein (16). The non-lectin tail also codes a Gly-Pro-Tyr-rich domain with a repetitive “PGAY” motif, assembling a collagen-like structure. The CRD domain forms a β-sandwich to bind complexes with oligosaccharides, poly-N-acetyllactosamine, galactomannan, and polymannan (17, 18).

Dimerization and alternative splicing of galectins can modify the biochemical properties of these proteins. Galectin-1 can form non-covalently linked homodimers through hydrophobic N- and C-terminal residues of two subunits interacting and associating by a 2-fold rotation axis vertical to the β-sheet plane. Although it was experimentally proven that only the dimeric form of galectin-1 is a biologically active form, monomers may also act in a limited fashion in certain signaling pathways as well (17, 18). Galectin-3 forms dimer and oligomer (pentamer) by using a repetitive PGAY motif (17). Galectin-7 forms a “symmetric” sandwich dimer by electrostatic interactions between the F-faces of two monomers. Galectin-7 and Galectin-2 can form “non-symmetric” dimers by electrostatic interactions between β-strand subunits (β1 and β6) of two monomers (4). Galectin-10 recognizes mannose residues rather than β-galactosides (19). Galectin-10 forms dimers between S-faces of two CRD domains of monomers, and mutation of Trp127 in this region could abolish dimerization (20). S-face to S-face dimer formation is unique among galectins, suggesting that galectin-10 may bind different ligands than other galectin isoforms (4, 20). Galectin-10 shares 6 of 7 residues involved in the recognition of glycans and its three-dimensional structure remains identical to other galectins (21). Galectin-13 forms dimers with a disulfide bridge between Cys 136 and Cys138 amino acid residue, and mutation of these two amino acids to serine could abolish dimerization (9, 22). Additionally, six hydrogen bonds, including amino acids of Val135, Val137, and Gln139 could stabilize dimer formation (23). Galectin-4, −6, −8, −9, and −12 form the group of tandem-repeat type galectins, in which CRD domains are connected by a linker peptide, whose length may vary. This enables galectins to crosslink glycan ligands to self-associate as dimers or oligomers (4).

## 3. Galectins in cancer-associated inflammation

### 3.1. Cancer-associated inflammation

Inflammation is closely associated with the progression of cancer and plays a pivotal role in tumor growth, metastasis, and drug resistance. Therefore, targeting inflammatory processes in cancer may represent an alternative therapeutic strategy (24). Tumor-extrinsic inflammation is triggered by many factors, including infection, obesity, autoimmune disorders, and exposure to tobacco and toxic substances (25). However, inflammation can also be induced by genomic mutation or genome instability of cancer cells, −called tumor-intrinsic inflammation-, that fuel immunosuppressive or tumor-promoting traits, leading to the recruitment and activation of inflammatory cells (25).

**Figure 2:**
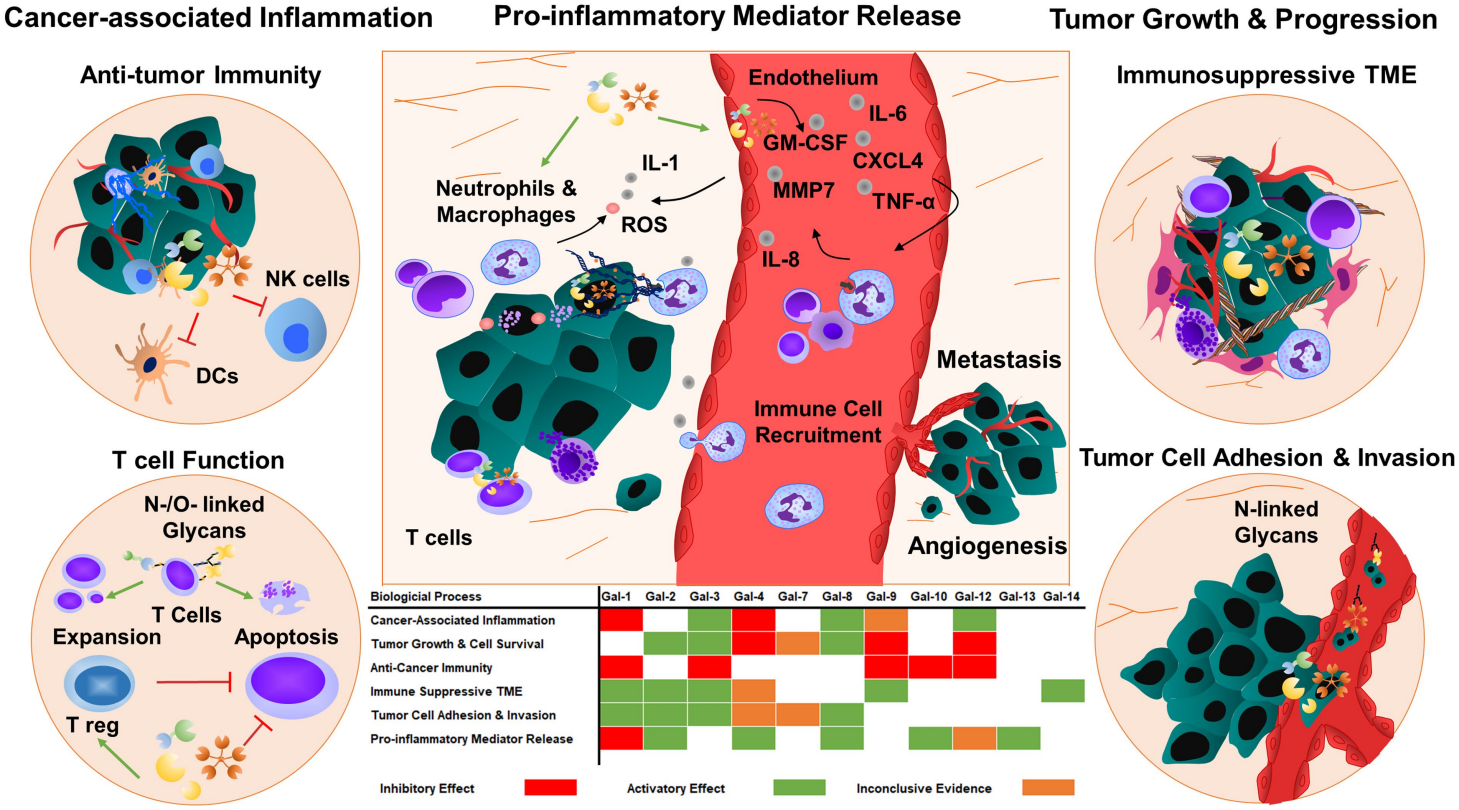
Galectins regulate tumor progression and metastasis by responding to inflammatory cues. Galectins can also prime tumor cells and tumor microenvironment to enhance tumor growth and angiogenesis by regulating cell adhesion, migration, and cytokine and chemokine release. The majority of galectins display pro-tumorigenic and metastatic functions, which promote immunosuppressive functions in immune cells and consequently inhibit anti-tumor immunity. TME: tumor microenvironment.

Glycosylation of cell surface proteins on cancer and immune cells is a dynamic process that affects cancer growth and inflammatory processes (26). The glycans decorate the cell surface of all mammalian cells and their structures can be altered during the course of cancer-induced inflammation. Multiple enzymatic processes generate glycosidic linkages of saccharides to other saccharides (26). Several factors and enzymes regulate the pathological modifications of these sugar trees during cancer cell differentiation and activation and are also influenced by inflammatory insults (26). Many of these processes are regulated by glycan-modifying proteins such as glycosyltransferases and glycosidases. The expression and enzymatic activity of these proteins depend on the cancer cell type, tumor microenvironment, and developmental stage of cancer. Several modifications are associated with tumor-mediated inflammatory responses such as the secretion and release of chemokines, cytokines, and growth factors (27). Galectins are the key players to regulate tissue homeostasis in cancer, thereby contributing to the progression of chronic inflammation in the tumor microenvironment and vasculature (Figure 2).

### 3.2. Galectin-mediated crosstalk between cancer and inflammation

#### 3.2.1. Galectin-1

Galectin-1 is synthesized by different cell types in the tumor microenvironment, including macrophages, regulatory T cells, dendritic cells, myeloid-derived suppressor cells, cancer cells, endothelial cells, and fibroblasts (28, 29). The contribution of galectin-1 to inflammation was initially described as a suppressor of cancer-associated inflammation. Glycans are found on many proteins on the immune cell surface such as CD43, CD45, CD69, pre-B cell receptor (BCR), and vascular endothelial growth factor 2 (VEGFR2), and changes in glycosylation modify immune cell responses. Galectin-1 recognizes lactosamines on N- and O-linked glycans and this interaction frequently induces T-cell apoptosis, which could be prevented by α2,6-linked sialic acid treatment (30). Galectin-1 is proposed as a negative regulator of the immune response since it can inhibit T cell-extracellular matrix (fibronectin and laminin) interactions thereby inhibiting the production of pro-inflammatory cytokines, released by activated T cells (31). Galectin-1 treatment can induce a shift from a Th1 towards a Th2-polarized immune response, characterized by decreased interferon gamma (IFNγ) and interleukin 2 (IL-2) secretion and increased IL-5 production, generated by lymph node cells (32–38). The inflammation-suppressing effect of galectin-1 was explained by the depletion of antigen-specific activated T cells through apoptosis (39). In line with these results, selective blockade of galectin-1 in tumor tissues resulted in increased Th1-mediated anti-tumor responses, suggesting potential involvement of this protein in tumor-immune escape (38). Furthermore, galectin-1 can mediate anti-tumor immunity by reducing transendothelial T cell migration (40). Galectin-1 function is associated with the presence of carcinoma-associated fibroblasts (CAFs) in solid tumors. In hepatocellular carcinoma (HCC) which is associated with chronic inflammation and fibrosis, galectin-1 deficiency with increased osteopontin and S100A4 expressions could amplify inflammatory processes, thereby exacerbating the liver injury and fibrosis in a mouse model of HCC (41). In galectin-1-deficient mice, high expression levels of chemokine ligand 5 (CCL5), C-C chemokine receptor 1 (CCR1), chemokine (C-X-C motif) ligand 2 (CXCL2), lymphotoxin beta receptor (LTBR), and serum amyloid A1 (SAA1) were detected with a robust infiltration of neutrophils and macrophages in the liver. Interestingly, galectin-1 effectively protects mice from acute liver inflammation in the C57BL/6 genetic background, but this was not observed in the FVB/NJ mouse strain implicating independent genetic factors (41). Although increased galectin-1 expression in HCC might inhibit fibrosis and inflammation, secreted galectin-1 promotes liver stellate cell activation and migration through clustering neuropilin-1/platelet-derived growth factor beta (NRP1/PDGFRβ) and NRP1/transforming growth factor beta receptor II (TGF-βRII) complexes (42). Therefore, disrupting glycosylation-dependent galectin-1/NRP1 interactions may provide new implications for the development of liver fibrosis therapy (42).

Activated stellate cells and cancer-associated fibroblasts, which represent most of the stroma cells in pancreatic ductal adenocarcinoma (PDAC), could develop an immunosuppressive microenvironment with increased numbers of regulatory T cells, but lack of effector CD4+/CD8+ T cells (43). Galectin-1 is mostly detected in the stroma of the pancreas, thereby promoting tumor-stroma crosstalk in PDAC. In pancreatic stellate cells and CAFs, knock-down of galectin-1 expression could impair fibroblast activation, cancer cell migration and invasion (44). Conversely, treatment of RWP1 pancreatic cells with recombinant galectin-1 could enhance the expression of several pro-inflammatory mediators, such as IL-1α, metalloproteinase 1 (MMP1), S100 calcium-binding protein A7 (S100A7) and ankyrin 3 (ANK3), thereby inducing metastatic potential (44).

In ovarian and breast cancers, IL-6-induced systemic inflammation enhances the mobilization of myeloid-derived suppressor cells which primed ɣδ T cells to produce galectin-1, thereby abolishing anti-tumor immunity (45). Galectin-1-mediated anti-tumor immunity is also regulated through its direct interactions with CD43, CD45, and CD7 receptors expressed on the surface of T cells, leading to the redistribution of glycoreceptors into segregated microdomains (46–48). Besides these mechanisms, glycosyltransferases can create N-acetyllactosamine ligands during cancer progression, which determine the susceptibility of T cells to cell death by galectin-1 (49). Galectin-1-induced apoptosis is regulated by several intracellular mediators, including transcription factors, caspases, cytochrome-C, and ceramide-associated signaling pathways in primary T cells. The pathological impact of galectin-1-induced T cell death was established using different disease mouse models. At lower concentrations, galectin-1 could effectively block T cell adhesion to the ECM, thereby abolishing pro-inflammatory cytokine secretions, such as tumor necrosis factor alpha (TNFα) and IFNγ, while at higher concentrations galectin-1 could induce T cell apoptosis (31). Blockade of immunosuppressive functions of galectin-1 induced tumor rejection, stimulating the generation of tumor-specific T-cell-associated response (31). Galectin-1 can also promote IL-10 production in T cells, suppressing Th1 responses (50). Galectin-1 also has a prominent expression in an immunosuppressive subset of dendritic cells, Zbtb46+, that infiltrate ovarian tumors (51). In melanoma cancer, galectin-1 had a substantial contribution to the immunosuppressive microenvironment, inducing apoptosis of cytolytic T cells and modulating the Th1-Th2 cytokine balance (38). In colon carcinoma, both tumor cell and stroma-resident galectin-1 could influence tumor growth by controlling the frequency and suppressive activity of CD8+ Treg cells (52). In line with this, high galectin-1 expression in colorectal adenocarcinoma patients was correlated to an elevated CD8+ Treg score and poor prognosis (52). In human chronic lymphocytic leukemia, galectin-1 is also expressed in nurse-like myeloid cells and macrophages, which induce the establishment of tumorigenic niches (53).

High levels of galectin-1 were found in the vascular endothelium of primary tumors in the lung, colon, head and neck, and oral cancers, and this high expression was usually associated with tumor-induced hypoxia, and angiogenesis (54). Consistently, knock-out of the galectin-1 gene in zebrafish and mouse models strongly impaired vascular guidance, tumor growth and angiogenesis (55). Knock-down of galectin-1 expression in hs683 glioblastoma cancer cells also inhibited angiogenesis by attenuating endoplasmic stress response and modulating expression of hypoxia-related genes such as CTGF, ATF3, PPP1R15A, HSPA5, TRA1, and CYR61 (56). Galectin-1 is upregulated on the endothelial cell surface after lipopolysaccharide (LPS) or cytokine treatment *in vitro* and increased galectin-1 expression of endothelial cells was found in inflamed lymph nodes (57). Galectin-1 expression is also enhanced in stromal and endothelial cells, which are treated by conditioned media derived from ovarian and prostate cancer cells (58, 59), indicating the role of galectin-1 in the crosstalk of tumor cells with inflamed vasculature.

#### 3.2.2. Galectin-2

Similar to galectin-1, galectin-2 can also bind T cells in a β-galactoside-specific manner and induce apoptosis (60). Elevated serum galectin-2 levels were detected in patients with colorectal and breast cancers. The pathological consequence of higher galectin-2 concentrations is to induce granulocyte colony-stimulating factor (G-CSF), IL-6, monocyte chemoattractant protein-1 (MCP-1) and growth-related oncogene alpha (GROα) secretion from endothelial cells, thereby increasing the expression of endothelial cell surface adhesion molecules, and enhancing cancer cell-endothelial cell interactions, angiogenesis and tumor metastasis (61). Higher galectin-2 levels are also a driving force of pro-inflammatory M1 phenotype in human monocytes by inducing toll-like receptor 4 (TLR4) signaling through CD14 interaction (62).

The role of galectin-2 in T cell apoptosis was studied in mice in which galectin-2 levels were inversely correlated to the occurrence of colitis. Mouse treatment with recombinant galectin-2 strongly reduced the rate of colitis by inducing T-cell apoptosis, located in the mucous membranes (63). In other studies, galectin-2 could interact with a pro-inflammatory cytokine lymphotoxin-α in smooth muscle cells and macrophages (64). Although several studies pointed out the pro-inflammatory and pro-angiogenic functions of galectin-2, experimental evidence is still missing to conclude the exact role of galectin-2 during cancer progression and metastasis.

#### 3.2.3. Galectin-3

Galectin-3 expression is upregulated in many types of solid tumors and increased protein levels correlate with the degree of malignancy (65). Galectin-3 levels in the blood are also increased in patients with breast, colon, and lung cancers (66). Interestingly, higher concentrations of galectin-3 were detected in metastatic tumors than in primary tumors (67). Galectin-3 is detected nearly in all the stages of tumor development (65, 67). Galectin-3 inhibits apoptosis by competing for a conserved structure with B-cell lymphoma 2 (Bcl-2) inhibiting the function of cell cycle inhibitors (68). Galectin-3 also enhances cell survival and proliferation by regulating phosphatidylinositol 3-kinase (PI3K)/protein kinase B (AKT) signaling and nuclear factor kappa B (NF-κB) pathways (69).

Galectin-3 induces secretion of IL-6, G-CSF, soluble intercellular adhesion molecule-1 (sICAM-1), and granulocyte-macrophage colony-stimulating factor (GM-CSF) in endothelial cells (70). These cytokines interact with the vascular endothelium and trigger diverse signaling pathways, thereby increasing the expression of endothelial cell surface markers and integrins, such as E-selectin, ICAM-1, and vascular intercellular adhesion molecule (VCAM-1) and αvβ1 integrin (70). In patients with metastatic colon cancer, higher serum levels of galectin-3 correlated with increased serum levels of G-CSF, IL-6, and sICAM1. In breast and colon cancer patients, increased serum levels of galectin-1 and galectin-3 also correlated with enhanced expression of G-CSF, and IL-6 (70), suggesting a modulatory role of galectin-3 in inflammation. Furthermore, neutralizing antibodies against pro-inflammatory mediators could prevent the adhesion of mucin-1 (MUC-1)-negative melanoma cells to the HMVECs endothelial cells, indicating that galectin-3-mediated secretion is regulated by inflammatory cytokines induces endothelium-tumor cell interaction (71).

Galectin-3 can directly interact with adhesion molecules. Galectin-3 binds the CD146 receptor on the surface of endothelial cells and consequently induces inflammatory cytokine secretion (72). Galectin-3-CD146 interactions could enhance endothelial cell migration (72). The oncofetal Thomsen-Friedenreich carbohydrate antigen (TF-antigen), Galβ1-3GalNAcα, is a pan-carcinoma antigen and highly expressed in almost all human carcinomas due to the aberrant glycosylation. Galectin-3 binds TF-antigen and this interaction induces diverse pathological processes such as tumor cell aggregation, cancer metastasis, and T cell apoptosis (73). Galectin-3 interacts with TF-antigen on the surface of the transmembrane mucin protein MUC-1 in cancer cells. The protein complex of Galectin-3-TF/MUC-1 induces MUC-1 cell surface polarization leading to the exposure of cell adhesion molecules, thus inducing tumor cell adhesion to the vascular endothelium which increases the number of cancer cell aggregates in the circulation (74). In contrast, O-glycan-modifying sialyltransferase ST6GalNAcs reduces the binding affinity of galectins (galectin-1 and galectin-3) to the tumor cell surface, thereby inhibiting intravascular aggregation of tumor cells and consequent metastasis (75, 76). Interestingly, liver macrophage-resident galectin-3 enhanced the liver retention of cancer cells that expressed high levels of sialyltransferase ST6GalNAc4, but low levels of glucosaminyltransferase GCNT3 (77).

Cancer stemness is a stem-cell-like phenotype of cancer cells and is involved in the reconstitution and propagation of tumor formation. Galectin-3 is upregulated in human renal cell carcinoma and associated with higher expression levels of stemness-related genes, such as Oct4, Sox2, and Nanog (78). In renal cell carcinoma, downregulation of galectin-3 could strongly inhibit cancer cell invasion, colony formation, sphere-forming ability and stemness-associated gene expression (78). In galectin-3 knock-down cells, CXCL6, CXCL7, and CXC chemokine receptor 2 (CXCR2) expressions were downregulated and overexpression of CXCR2 could restore the ability of galectin-3 knock-down cells to form spheres (78). Altogether, these results suggest that galectin-3 regulates cancer stemness, and induces cancer-cell intrinsic inflammation by upregulating CXCR2, thereby increasing tumor progression.

The function of Galectin-3 is also associated with RAS signaling in cancer since the CRD domain binds and stabilizes the active conformation of KRAS (79, 80). Galectin-3 with KRAS can induce PI3K activation as well as constitutive activation of the RAF/MEK/ERK signaling cascade, thereby regulating tumor cell functions. Moreover, in glioblastoma cancers, galectin-3 favors tumor invasiveness through a micropinocytosis-mediated uptake mechanism (80). Upregulation of galectin-3 expression was correlated with increased anchorage-independent growth and organ colonization. Galectin-3 potentiates cell migration and metastasis through activation of the K-RAS–RAF-Erk1/2 pathway in colon cancer. Galectin-3 also regulates pancreatic cancer metastasis through the activation of RAS–ERK/AKT and Rel-A signaling pathways, thereby increasing cell migration, and survival (81).

Downregulation of galectin-3 could effectively inhibit tumor cell migration, invasion, cell proliferation and metastasis in osteosarcoma, thyroid, and gastric cancer (82–84). Interestingly, galectin-3 knock-down studies published by Bresalier et al. showed direct evidence of its role in tumor invasion and metastasis. The authors observed reduced liver colonization and spontaneous metastasis of galectin-3 knock-down LSLiM6 and HM7 cells, two derivatives of the colonic adenocarcinoma LS174T cells with high liver-metastasizing potential (85). Consistently, knock-down of the galectin-3 gene was associated with the inhibition of cell migration, invasion, cell proliferation, colony formation and tumor growth in nude mice (85). Galectin-3 expression in PC3 human cancer cells resulted in cell cycle arrest at the G1 phase; upregulation of p21 levels in nuclei, and hypophosphorylation of the tumor suppressor protein pRb (86). In mouse models of breast and prostate cancers, galectin-3 was cleaved by MMP2 and MMP9 and this was associated with enhanced tumor growth and angiogenesis (87, 88). In line with this result, the cleavage products of galectin-3 were detected in the blood serum of prostate cancer patients with advanced or metastatic tumors (86, 89).

Galectin-3 is also involved in tumor immunity to regulate natural killer (NK) and T cell functions. Galectin-3 could inhibit the interaction of NK cells with cancer cells, thereby evading the ability of cytotoxic effects of NK cells to kill them (90). Extracellular galectin-3 binds several glycoproteins on the T cell surface and induces apoptosis (91). Galectin-3 also suppresses CD8 T cell function in melanoma cancer, possibly influencing lymphocyte activation gene-3 (LAG-3) function on the immune cell surface (92). Furthermore, increased galectin-3 expression is associated with the loss of T cell receptor (TCR) and CD8 marker localization and loss of effector T cell function (93, 94). Galectin-3 could induce a robust production of pro-inflammatory cytokines in different immune cells (95). In line with this, *in vivo* depletion of galectin-3 was shown to increase both the number of functional CD8+ T cells and the consequent expression of pro-inflammatory cytokines, thereby inducing tumor rejection in galectin-3-deficient mice (92, 94).

Galectin-3-mediated ligand clustering triggers neutrophils to phagocytose, produce reactive oxygen species (ROS), release proteases, and secrete IL-8 (96–98). Galectin-3 induces degranulation in mast cells. Recent studies revealed the critical roles of galectin-3 in this cell type since galectin-3-deficient mast cells show reduced histamine release and IL-4 secretion (99).

#### 3.2.4. Galectin-4

Downregulation of galectin-4 was observed in acute myeloid leukemia and colon cancer (100, 101). Galectin-4 was proposed as a tumor suppressor inhibiting cell proliferation by down-regulating wingless/integrated (Wnt) and IL-6/NF-κB/Signal transducer and activator of transcription 3 (STAT3) signaling that balance epithelial cell homeostasis in the intestine (102). Galectin-4 also inhibits cell migration and metastasis in PDAC (103). In this pathological condition, galectin-4 markedly reduced cytoplasmic β-catenin levels, counteracted Wnt signaling function, and rendered pancreatic cancer cells sensitive to Wnt inhibitors (103). Consequently, the galectin-4 deficiency was associated with early cancer recurrence and death, defined as occurring twelve months after curative surgery. Under inflammatory conditions, galectin-4 suppresses tumor growth by stimulating memory CD4+ T cell expansion (104, 105). This process involves the interaction of galectin-4 with immature core-1-expressing O-glycans generated by downregulation of the core 2-β1,6-N-acetylglucosaminyltransferase-1 (104). Consequently, ectopic expression of core 2-β1,6-N-acetylglucosaminyltransferase-1 could reduce tumor growth (104).

In sharp contrast with these studies, the galectin-4 function was also proposed to enhance tumor angiogenesis and metastasis (61, 100, 106). Upregulation of galectin-4 was observed in patients with advanced liver cancer, intraductal breast cancer, and gastric and colorectal cancers. Elevated galectin-4 expression was associated with venous but not lymphatic invasion of lung adenoma cancer cells (107). Therefore, quantification of galectin-4 expression levels in the blood could serve as an oncogenic biomarker. Galectin-4 enhanced cancer cell adhesion to the endothelial cells through binding with TF-antigen on cancer-associated MUC-1 (108).

Furthermore, galectin-4 regulates the pathogenesis of inflammatory bowel diseases and colitis. Galectin-4 activates CD4+ T cells and IL-6 secretion that contributes to the progression of colitis in mice. This disease was cured by injecting anti-galectin-4 blocking antibodies into mice (109). Galectin-4 directly interacts with the immunological synapse of CD4 + T cells thereby activating protein kinase C (PKC) signaling which further stimulates IL-6 production, thereby exacerbating inflammation in the intestine (109).

#### 3.2.5. Galectin-7

Galectin-7 was described as an epithelial differentiation marker in different organs (110). High expression levels of galectin-7 were found in aggressive subtypes of breast cancer, frequently with a basal-like phenotype and estrogen receptor-negative tumors (111). Galectin-7 also enhances spontaneous metastasis in both human epidermal growth factor receptor 2 (HER2) overexpressed and basal-like lineages of breast cancer (112). Upregulation of galectin-7 was sufficient to induce a metastatic feature of tumor cells and rendered them resistant to cell death (112). Galectin-7 was detected in the cytoplasm, but it can translocate into the nuclei after cellular stimuli or also be secreted into the extracellular place (113). Galectin-7 gene expression is modulated by several cytokines, transcription and growth factors. P53-induced galectin-7 expression in breast cancer cells correlated with high NF-κB activity, suggesting that induction of galectin-7 expression by P53 is dependent on NF-κB (114). In addition, NF-κB can bind galectin-7 promoter, indicating that galectin-7 and P53 may regulate cancer metastasis through common mechanisms (114). In galectin-7-transfected urothelial cancer cells, increased ROS production and jun N-terminal kinase JNK/ BCL2-associated x protein (Bax) signaling were detected (115). Galectin-7 expression was strongly enhanced by insulin growth factor 1 (IGF-1) and cyclooxygenase-2 (COX-2) in melanoma (116, 117). Although galectin-7 rendered B16F1 melanoma cells resistant to apoptosis but also inhibited cancer cell motility through increased expression of early growth response protein 1 (EGR-1) (118). It was proposed that the upregulation of galectin-7 expression contributes to cell survival by enhancing extracellular signal-regulated kinase (ERK) and Jnk signaling pathways (119). Pro-invasive and pro-metastatic functions of galectin-7 were associated with increased expression of MMP2 and MMP9 in oral squamous cells and ovarian carcinoma (119). Galectin-7 binds human tumorous imaginal disc (Tid1) heat shock protein 40 (Hsp40) and this interaction attenuates tumorigenicity and metastasis of head and neck squamous sarcoma cancer cells (120). Interestingly, N-linked glycosylation of Tid1 is required to interact with galectin-7, which induces protein degradation of galectin-7, explaining the protective effect of this molecular interaction (120).

Galectin-7 is secreted by ovarian cancer cells and is involved in the regulation of tumor invasiveness. Recombinant human galectin-7 killed Jurkat T cells and human peripheral T cells, suggesting that galectin-7 has strong immunosuppressive properties (121). Constitutive expression of galectin-7 was found in aggressive metastatic lymphoma. Transfection of T-cell lymphoma cells with galectin-7 also increases tumor growth in mice (122). The histopathological analysis of mice showed large metastatic tumors with increased invasive and infiltration rates (122). Although galectin-7 can act as an oncogene, the opposite roles have been also shown in gastrointestinal cancers. Ectopic expression of galectin-7 in colon cancer cells rendered them more sensitive to apoptosis after treatment with actinomycin D, cobalt chloride, hydrogen peroxide or under hypoxia (123). Consequently, overexpression of galectin-7 strongly reduced gastric cancer cell proliferation, migration, and invasion. Interestingly, lower galectin-7 expression was detected in patients with gastric cancer compared to controls and the expression levels were significantly associated with tumor grade, stage, and better survival of patients with gastric cancer (123). Galectin-7 also reduces the invasiveness of prostate cancer cells by inhibiting cell motility and rendering cancer cells sensitive to apoptosis in response to chemotherapeutic agents (124). Interestingly, the CRD-defective mutant form of galectin-7 (R7S) can modulate apoptosis or translocate to the mitochondria and nucleus, indicating functional independency from its CRD domain (124). However, the CRD domain was necessary to inhibit the invasive behavior of cancer cells thereby potentiating tumor growth.

#### 3.2.6. Galectin-8

Galectin-8 binds different types of integrins (α1β1, α3β1, α5β1 and α6β1) with similar kinetics to fibronectin, but not interacts with α2β1 or α4β1 integrins, suggesting an important but selective function in cell spreading and adhesion (125). Galectin-8 and integrin interaction creates a complex structure, involving sugar/protein interactions, which regulates actin cytoskeletal rearrangements. After integrin activation with galectin-8, phosphorylation of paxillin, focal adhesion kinases, and activation of PI3K, Rac family small GTPase 1 (Rac-1) and ERK1/2 signaling was detected (126, 127). Galectin-8 can enhance tumor metastasis by regulating the rearrangement of the cytoskeleton and E cadherin expression, inhibiting anoikis and homotypic aggregation of cancer cells (128). Galectin-8 is highly expressed in breast, prostate, and lung cancer tissues and elevated serum levels of galectin-8 promote cellular interactions between cancer cells and vascular endothelium (129). The treatment of endothelial cells with galectin-8 induces the production of several inflammatory cytokines such as IL-8, RANTES/CCL5, GRO-α, GRO-γ, M-CSF, IL-6, and MCP1 (130). Galectin-8 also stimulates cytokine expression in other cell types such as liver, lung, spleen, osteoclasts, and bone marrow-derived dendritic cells (129). Interestingly, metastasis-promoting effects of galectin-8 are independent of endothelial cell interaction. Galectin-8-linked tumor invasion and metastatic tumor cell spread are also regulated by immunomodulatory cytokines and chemokines (129).

#### 3.2.7. Galectin-9

Galectin-9 is constitutively expressed in antigen-presenting cells and its expression is upregulated by interferons in cancer cells (131). The function of galectin-9 is strongly associated with tumor-immune microenvironment and immunosuppression in different cancer types. Galectin-9 binds to Ig and mucin domain-containing molecule 3 (Tim-3) on the surface of T cells, thereby inducing cell death (132–134). Besides, galectin-9 also regulates immunosuppressive immune cell function by promoting regulatory T-cell differentiation and expansion through binding with a cluster of differentiation 44 (CD44) (132). Although the galectin-9 expression is associated with a good prognosis in some cancers, it is associated with unfavorable outcomes in other tumor types (135). Galectin-9 is strongly upregulated in the most invasive type of multiple glioblastomas and is correlated with poor patient survival (136). It was proposed that the blockade of galectin-9-mediated Tim3 signaling is effective to impair glioma progression by inhibiting macrophage M2 polarization and tumor angiogenesis (137). Interestingly, endothelial cell activation and angiogenesis can regulate the alternative splicing of galectin-9 gene, indicating that the pathophysiological change of the tumor microenvironment could induce epigenetic factors, thereby changing the alternative splicing mechanism, protein structure and function of galectin-9 variants (138).

Upregulation of galectin-9 expression was found in immune cells, tumor cells, and blood plasma of PDAC and melanoma patients (139, 140). Furthermore, elevated galectin-9 levels were detected in tumor-infiltrating T lymphocytes from non-responders to anti-programmed death-1 (PD-1) therapy compared with those from the responders (131). In a mouse model of PDAC, blockade of galectin-9 function could effectively retard tumor progression and prolonged survival by inducing maturation and expansion of macrophages and dendritic cells, and modulating the immune response of NK and tumor-associated CD8+ T cells (141–143). In the mouse models of triple-negative breast cancer, 4-1BB, a galectin-9-neutralizing antibody in combination with an agonist antibody to the tumor necrosis factor receptor (TNFR) synergistically inhibited tumor growth and prolonged mouse survival (144).

Galectin-9 interacts with cell surface adhesive molecule CD44 and this interaction inhibits the complex formation of CD44 with hyaluronic acid, which consequently attenuates metastatic dissemination of melanoma and colon cancer cells (145). Similarly, galectin-9 also acts as a competitive inhibitor by blocking the VCAM1-α4β1 interactions, thereby reducing tumor cell adhesion to the vascular endothelium and consequent tumor cell extravasation and metastasis (145). Furthermore, galectin-9 can inhibit melanoma cancer metastasis by triggering the aggregation of cancer cells, which impairs cell detachment and escape from the primary tumor (146). Galectin-9 is also involved in epithelial cell polarity through binding with apically residing glycolipid (Forssman antigen) (147). The pro-apoptotic function of galectin-9 has been described in ovarian cancer, leukemia, and myeloma cell culture models (148–150). Galectin-9 inhibited the growth of leukemia and myeloma cancer cells through activating transcription factor-ATF-Noxa, JNK, P38 mitogen-activated protein kinase (MAPK) and caspase-3 pathways (149, 150). Altogether, these studies suggested that depending on the disease context, galectin-9 can serve as a biomarker and therapeutic target in cancer.

#### 3.2.8. Galectin-10

Galectin-10 is highly expressed in human eosinophilic and basophilic granulocytes, but not in mouse granulocytes (151–153). Interestingly, a subgroup of eosinophils was identified with high levels of galectin-10 and CD16 expression, and these eosinophils are more potent T-cell suppressors than conventional eosinophils (154). Proteomic analysis of human CD4+ and CD25+ regulatory T cells identified galectin-10 as a novel marker that distinguishes this population from resting and activated CD4+ T cells (155). Inhibition of galectin-10 restored the proliferative capacity of human Tregs and abolished their immunosuppressive function (155).

#### 3.2.9. Galectin-12

Galectin-12 deficiency leads to M2 macrophage polarization that consequently results in reduced foam cell formation and pro-inflammatory cytokine production (156). Galectin-12 also affects myeloid differentiation, which is associated with chemotherapy resistance (157, 158). Overexpression of galectin-12 induces cell cycle arrest at the G1 phase in cancer cells, thereby suppressing cell proliferation (159). A positive association between cell differentiation and galectin-12 expression was described in human colorectal cancer cell lines (160).

#### 3.2.10. Galectin-13

Galectin-13 is predominantly detected in the placenta (161). It is also expressed in the kidney, bladder, and spleen and also in neurogenic tumors, liver adenocarcinoma, and melanoma (162). Placental galectin-13 was shown to enhance the apoptosis of T cells, by inducing the synthesis of IL-8 (163). Furthermore, T cells produce a high amount of chemotactic molecules, thereby inducing neutrophil extravasation in the endometrial decidua (164). Interestingly, galectin-13 can also trigger necrosis to induce the recruitment of immune cells, thereby allowing trophoblast invasion and vessel remodeling (165). Galectin-13 also polarizes placental neutrophils toward an immune regulatory phenotype by triggering the production of ROS, hepatocyte growth factor (HGF), and MMP9 and upregulating the expression of programmed death-ligand 1 (PD-L1) (166). Future studies are required to study the role of galectin-13 in the regulation of cancer-associated immunosuppressive and inflammatory phenotype.

#### 3.2.11. Galectin-14

Galectin-14 is expressed in the placenta. It was proposed that galectin-14 may regulate immune tolerance at the maternal-fetal interface by inducing apoptosis of leukocytes and T cells (163). Galectin-14 also facilitates migration and invasion by promoting an epithelial-mesenchymal transition (EMT)-like phenotype in trophoblasts (167). Galectin-14 is expressed at a high level in several cancer types, including liver, breast, uterine, and ovarian cancer (168). Furthermore, increased expression of galectin-14 has been reported to correlate with shorter survival in high-grade serous adenocarcinoma ovarian cancer (168).

## 4. Galectins in thrombosis and thromboinflammation

### 4.1. Thrombosis and thromboinflammation

Besides the regulation of the immune system, galectins also regulate hemostasis and thrombosis through interplay with platelets, endothelium, and the coagulation system. Upon vessel injury, subendothelial matrix proteins, such as collagen are exposed to the blood flow, anchoring von-Willebrand-Factor (vWF) and initiating platelet glycoprotein (GP)Ibα–vWF interaction and subsequent GPVI–collagen interaction, a crucial step in platelet activation (169). Activated platelets express several integrins on the surface. The outside-in activation of integrins with their extracellular ligands also induces platelet adhesion to the injured vessel wall. In addition, activated platelets release many bioactive molecules and secondary mediators, such as fibrinogen (FGN), vWF, adenosine diphosphate/adenosine triphosphate (ADP/ATP), and serotonin from their alpha (α) and dense delta (δ) granules further enhancing the pro-thrombotic process to induce the recruitment of circulating platelets to the growing thrombi. After platelet accumulation, the blood coagulation pathway induces the second wave of hemostasis, generating thrombin through extrinsic and intrinsic pathways. In turn, thrombin converts soluble fibrinogen to fibrin, resulting in enhanced platelet activation (169, 170). Activated platelets expose phosphatidylserine (PS) on their surface, which facilitates the binding of coagulation factors that stimulate the generation of thrombin in other circulating and vascular cells. These processes can also occur in diseased vessels, such as atherosclerotic inflamed vessels as well as when cancer cells grow and or propagate to the organism, forming metastases (171).

**Figure 3:**
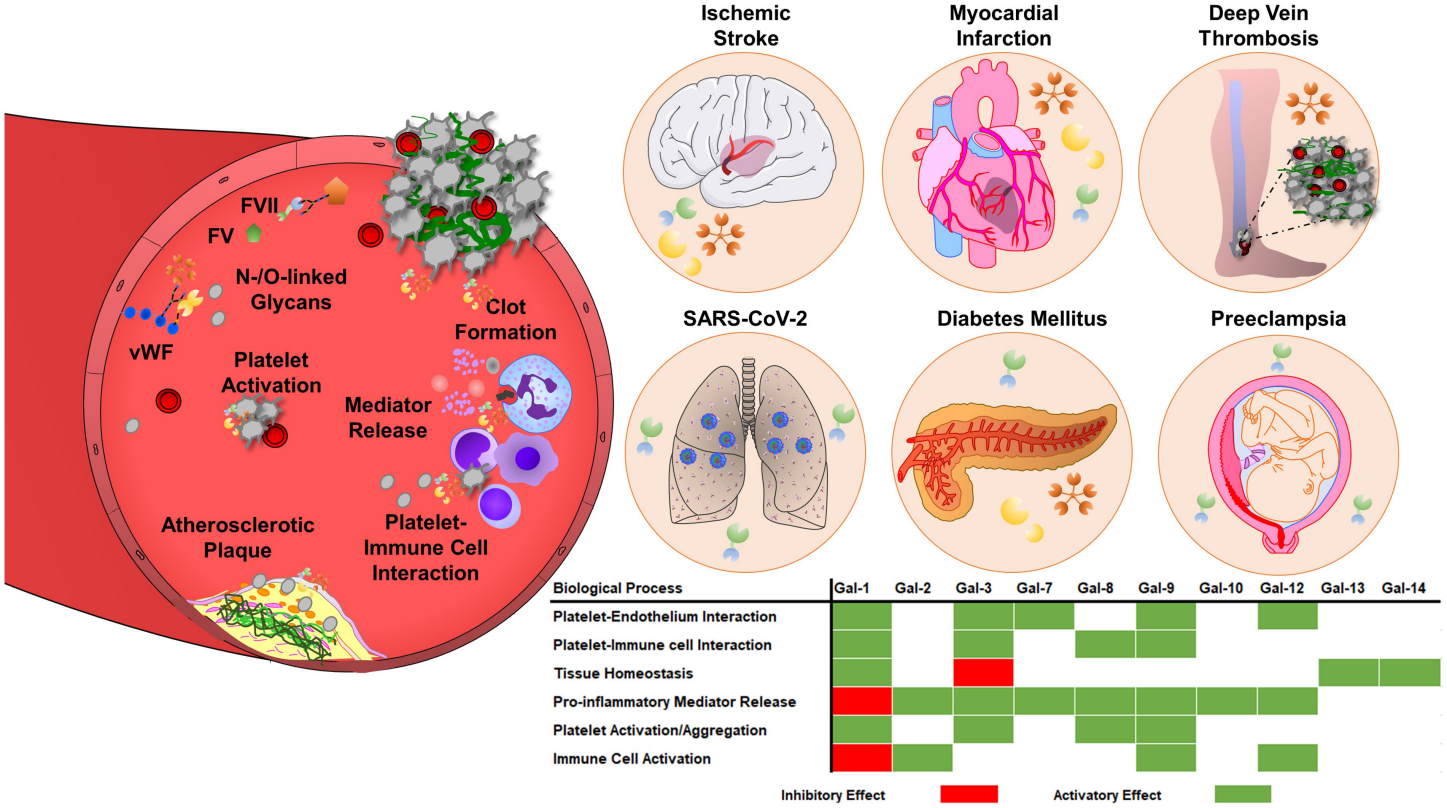
Galectins are involved in thrombosis and thromboinflammation through interplay with vascular endothelium, immune cells, and blood platelets. Galectins can promote platelet adhesion activation and regulate coagulation pathways, through interactions with glycoproteins, integrins, and coagulation factors. Galectins have multiple roles in thromboinflammatory diseases, such as atherosclerosis, myocardial infarction, deep vein thrombosis, or ischemic stroke. The regulatory role of galectins strongly depends on the pro-thrombotic, pro-inflammatory, and immunosuppressive landscape of the vasculature and surrounding environment. Therefore, the effects of galectins may vary depending on the stage and pathogenesis of the disease.

The simultaneous activation of thrombotic and inflammatory responses occurs in various diseases and has been referred to as thromboinflammation (172, 173). Platelets and the intravascular thrombus formation represent a scaffold for the cells of the innate immune system to facilitate the recognition, containment, and destruction of pathogens (174, 175). Innate immune cells and platelets interact and reciprocally influence the thrombotic potential and immune reaction. Neutrophils interact with P-selectin expressed on the surface of activated platelets via P-selectin glycoprotein ligand-1 (PSGL-1) (176). Platelet interaction enhances downstream MAPK activation in neutrophils, IL-8 production, and neutrophil adhesion to fibrinogen by Src kinase-dependent activation of β2 integrin, thereby facilitating increased immunogenic potential (177). Platelet GPIbα binds myeloid cells via various expressed receptors like integrin αMβ2 or complement receptor 3 (CR3), promoting leukocyte recruitment and activation, as well as integrin-mediated adhesion and diapedesis, that result in the infiltration of inflammatory cells into the tissues (178). Galectin levels are dramatically increased in cardiovascular diseases, including atherosclerosis, diabetes mellitus, ischemic stroke, and venous thromboembolism (Figure 3), suggesting a pathological link between galectin function and thromboinflammation (10).

### 4.2. Galectin-mediated crosstalk between thrombosis and inflammation

#### 4.2.1. Galectin-1

Two isoforms, galectin-1 and galectin-3 can bind N- and O-glycans on the surface of coagulation factor VIII (FVIII), indicating an important role of these interactions in the regulation of the coagulation cascade (179). In normal physiological conditions, plasma contains 1–10 μg/ml galectin-1 and galectin-3 and their levels are stabilized through binding to vWF (179, 180). In the absence of galectin-1 and galectin-3, a strong accumulation of platelet-decorated vWF strings was observed on the surface of endothelial cells, subsequently enhancing the formation of arterial thrombi (181). Galectin-1 also regulates fibrinolysis through binding to tissue-plasminogen activator (t-PA) and increasing its catalytic activity (182). Galectin-1 can directly activate platelets. Pre-incubation of human platelets with exogenous galectin-1 enhanced integrin αIIbβ3 activation, P-selectin exposure, and microparticle shedding (183). Galectin-1-deficient (*Lgals1^−/−^*) mice displayed normal platelet count, but increased bleeding time, indicating altered hemostasis (183). Biochemical analysis using integrin αIIbβ3-deficient platelets, isolated from patients with Glanzmann’s thrombasthenia, identified galectin-1 as a functional binding partner of integrin αIIbβ3 (183). Although normal levels of integrin αIIbβ3 were observed in *Lgals1^−/−^* platelets, impaired spreading on fibrinogen and reduced clot retraction were observed, indicating a failure in the “outside-in” activation of αIIbβ3 signaling. Galectin-1 was proposed as a prominent factor linking thrombosis and inflammation (183). Galectin-1-stimulated platelets expose P-selectin which interacts with PSGL on leukocytes, thereby inducing the formation of heterotypic aggregates (183).

Although galectin-1 is detected in normal heart tissues including cardiomyocytes, its level is strongly increased during acute myocardial infarction (184). It was hypothesized that early upregulation of galectin-1 can serve as a safeguard mechanism to prevent dysregulated inflammatory responses (184). Elevated galectin-1 expression influences the resolution of inflammation at later steps of acute myocardial infarction by restoring tissue homeostasis. In line with these assumptions, *Lgals1^−/−^* mice displayed adverse ventricular remodeling after acute myocardial infarction associated with excessive inflammation (184). Galectin-1 deficiency increased cardiac infiltration of T cells, macrophages, and NK cells, but reduced recruitment of anti-inflammatory Treg cells, indicating that galectin-1 regulates Treg-mediated protection during acute myocardial infarction (184). Protective effects of galectin-1 were also observed in the early phase of ischemia–reperfusion-induced kidney injury, improving renal function and inhibiting the release of pro-inflammatory mediators and leukocyte transmigration (185).

Galectin-1 is expressed in neurons and glial cells. Galectin-1 stimulates astrocyte differentiation and secretion of brain-derived neurotrophic factor (186). The function of galectin-1 was studied in an experimental stroke model and elevated galectin-1 expression was detected in the infarcted and penumbra area (187). After ischemic insult in stroke, galectin-1 treatment sustained neurogenesis in a carbohydrate-dependent manner and this process was inhibited by an anti-galectin-1 blocking antibody (187). The transfer of galectin-1-overexpressing stem cells into the mice strongly reduced infarct volume upon ischemic stroke. Galectin-1 treatment also showed neuroprotective effects by inhibiting central nervous system inflammation and preventing inflammation-induced neurodegeneration (186). Proposed mechanisms involved galectin-1 binding to M1-type microglia, sloping the balance toward the M2-type anti-inflammatory phenotype (186). Furthermore, galectin-1 protected neurons by modulating the expression of the glutamate receptor NMDA, which prevented the neurotoxicity of glutamate during the stroke (188). Although increased serum galectin-1 levels were observed after ischemic stroke, no association has been observed between galectin-1 levels and post-stroke recovery (189).

#### 4.2.2. Galectin-2

Macrophages and smooth muscle cells of atherosclerotic lesions express both galectin-2 and lymphotoxin-α, indicating the involvement of galectin-2-mediated pro-inflammatory cytokine responses in this process (64). Galectin-2 acts as a pro-inflammatory factor by transforming human macrophages toward an M1 phenotype through binding to CD14 and activating the TLR4 pathway, thereby inhibiting arteriogenesis (62). Studies on the Japanese population showed the existence of a functional single-nucleotide polymorphism (SNP) rs7291467 (C3279C-T) in the galectin-2 coding gene that was strongly associated with myocardial infarction (64). C3279C-T genetic substitution affected the transcriptional levels of galectin-2 *in vitro*, altering the secretion of lymphotoxin-α and inflammation (64), indicating on the link between galectin-2 function and pathogenesis of myocardial infarction.

#### 4.2.3. Galectin-3

Atherosclerotic plaques express high levels of galectin-3 and correlate with the degree of atherosclerosis and inflammation (190). Galectin-3 exacerbates vascular inflammation by stimulating macrophages to produce a range of pro-inflammatory mediators (191). Galectin-3 is also associated with macrophage differentiation absorbing oxidized low-density lipoprotein (ox-LDL) and transforming macrophages into foam cells, thereby accelerating atherosclerosis (192). Galectin-3 aggravates the ox-LDL-mediated endothelial injury by stimulating inflammation through the activation of integrin β1-RhoA-JNK signaling (193). Galectin-3 inhibition was shown to reduce the pathological consequences of a high-fat diet-induced cardiac lipotoxicity in mice (194). Galectin-3 also activates fibroblasts and contributes to the synthesis of collagen I, additionally modulating the ECM degradation through tissue inhibitor of metalloproteinase (TIMP) and MMP activation (195–197). Galectin-3 strongly binds the immunoglobulin E (IgE) receptor and this interaction triggers basophil and mast cell activation, thereby enhancing inflammatory processes (198).

Using mouse stasis models of venous thrombosis, galectin-3 and its binding protein (Gal3-BP) were detected on vein walls, red blood cells, platelets, and microparticles, contributing galectin-3 functions to venous thrombosis (180). In prospective epidemiological studies, participants with plasma galectin-3 concentrations in the highest quintile had a higher risk of venous thromboembolism than did those in the lowest quintile. Gal3-BP was also detected on leukocyte-derived microparticles during deep venous thrombosis (180). Consistently, galectin-3 and Gal3-BP-deficient mice showed significantly reduced deep venous thrombosis. Interestingly, a similar phenotype was observed in IL-6-deficient mice (180). Although the galectin-3 treatment could restore thrombus formation in galectin-3-deficient mice, it had no effect in IL-6-deficient mice, suggesting that galectin-3 may promote deep venous thrombosis through upstream signaling of IL-6 pathway (180). A direct correlation between blood plasma levels of galectin-3/Gal3-BP and pro-inflammatory cytokines and chemokines was observed in patients with deep venous thrombosis (180). Galectin-3 treatment in mice or cell cultures could induce IL-6, CCL2, and TNF-α secretion in a dose-dependent manner (180).

During venous thrombosis, red blood cells promote thrombus growth by enhancing platelet recruitment, subsequent blood coagulation, and fibrin formation (174). Platelet GPVI contributes to deep venous thrombosis by interacting with fibrin (199). Galectin-3 is highly expressed in red blood cells, vessel walls, and leukocytes (180), and can interact with platelet GPVI, indicating that this interaction may regulate both thrombosis and thromboinflammation in different vascular territories. Recently we showed that galectin-3 is a binding partner of platelet GPVI and that interaction induces platelet activation, shape change, degranulation, and ATP release, thereby inducing tumor metastasis (200).

High plasma galectin-3 levels participate in cardiovascular disease by directly enhancing endothelial cell injury, platelet activation, and immune cell migration. High serum and plasmatic galectin-3 levels are also associated with the severity of coronary stenosis in patients with coronary artery disease (201, 202). Accumulated galectin-3 directly activates the dectin-1 receptor, thereby potentiating platelet aggregation, and ATP release in the blood, which contributes to the development of thrombosis and myocardial infarction (201). Galectin-3 has a pivotal role in the regulation of immunological functions in dendritic cells, monocytes, and macrophages (95). Galectin-3 stimulates the expression of pro-inflammatory chemokines to attract monocytes and macrophages to the vascular injury sites (191). Galectin-3 can also bind mucins and integrins at the cell surface thereby promoting adhesion between granulocytes and vascular endothelium (203). Inhibition of galectin-3 function strongly reduces expression of pro-inflammatory mediators, such as IL-6, IL-1β, IL-23, and P19, and upregulates IL-10, IL-12, TLR/NLR-pathways in dendritic cells and monocytes, thereby inhibiting the development of Th17/T2 cells and innate immunity (204). Plasma galectin-3 levels are increased in patients with peripheral artery disease and are positively correlated with oxidative stress markers, such as F2-isoprostanes (205). Interestingly, oxidative stress increases galectin-3 levels in monocytes (206). In activated neutrophils, galectin-3 enhances the secretion of hyperoxides (205). Oxidative stress-promoting effects of galectin-3 were also observed in mast cells, this was blocked by an antioxidant superoxide dismutase. High galectin-3 expression was detected in M1 macrophages in pancreatic inflammatory infiltrates in a model of type 2 diabetes (207). Galectin-3 also acts as an endogenous paracrine ligand of TLR4 on microglia cells, inducing M1-type phenotype exacerbating inflammation (208).

The role of galectin-3 in ischemic stroke remains controversial. In the rat middle cerebral artery occlusion (MCAO) model of ischemic stroke, galectin-3 promotes neuronal cell viability during oxygen–glucose deprivation conditions by increased phosphorylation of AKT, decreased phosphorylation of ERK1/2 and impaired activation of caspase-3 (209). Galectin-3 supports neuro-vascular protection and functional post-stroke recovery by modulating angiogenic and apoptotic pathways in the brain (209). In mouse models of hypoxic–ischemic brain injury, galectin-3 deficiency reduces oxidative stress, MMP activity, and overall brain injury. Recently the studies by Zhuang et al., which included 288 stroke patients showed that higher serum levels of galectin-3 were associated with stroke severity at admission and stroke prognosis at the discharge of ischemic stroke (210). In another study, higher levels of serum galectin-3 were independently associated with an increased risk of death or major disability after stroke onset (210).

#### 4.2.4. Galectin-4

Obesity is strongly associated with the progression of cardiovascular diseases and diabetes. Elevated galectin-4 levels were associated with a higher probability of obesity-associated hospitalization in diabetic patients (211). Thus, galectin-4 could be a risk factor in the pathogenesis of diabetes mellitus.

#### 4.2.5. Galectin-7

Galectin-7 is predominantly expressed in stratified skin epithelium and upregulated during skin injury (113). Galectin-7-deficient mice showed delayed wound healing which is caused by impaired cicatrization (113). Interestingly, the process of wound healing was attenuated by lactose, but not sucrose, indicating a selective, lactose-dependent involvement of galectin-7 in this process (113). Upregulation of galectin-7 was observed during placenta formation in mice that induce systemic preeclampsia via impaired placental formation and enhanced release of inflammatory cytokines (212). These studies highlighted galectin-7 as a sentinel molecule, constantly sensing the integrity of the tissues under a steady state while contributing to posttraumatic tissue generation. Disrupted integrity of epithelium and elevated galectin-7 levels were usually observed in asthma and chronic obstructive pulmonary disease. In these pathological conditions, silencing of galectin-7 expression could inhibit TGF-β-induced apoptosis in airway epithelium through alteration of the JNK-pathway (213, 214).

Galectin-7 can bind to red blood cells, thereby recognizing blood group-containing antigens (215). Diverse microorganisms such as *Escherichia coli*, *Klebsiella pneumoniae*, *Providencia alcalifaciens*, and *Streptococcus pneumoniae* express blood group-like antigens, allowing them to act as invading pathogens and induce injury in host cells (215). Interestingly, galectin-7 can recognize these pathogens, each of which uses molecular mimicry while failing to induce host cell injury, thereby providing an innate form of immunity (215). Galectin-7 also regulates CD4+ T cell immunity, by promoting the proliferation and polarization of Th1/2 cells, balancing toward the Th1 phenotype, which can potentially induce autoimmune diseases (216).

#### 4.2.6. Galectin-8

Platelets present two splice variants of galectin-8 with variable linker regions that are exposed on the platelet surface after thrombin stimulation (217). Using mass spectrometric and biochemical analysis it has been shown that galectin-8 binds αIIbβ3 integrin on the platelet surface, indicating an important function in platelet adhesion and spreading (217). Soluble galectin-8 binding to integrin modulates the conformational change of integrin to a high-affinity state, thereby opening the molecular surface for fibrinogen binding (217). Using platelets isolated from Glanzmann’s thrombasthenia patients with αIIbβ3 integrin deficiency, functional studies showed a reduction of galectin-8 binding on the platelet surface (217). Moreover, galectin-8 could activate platelets through Src, PI3K/Akt, and PLCγ2 signaling pathway (217).

Factor V (FV) is not synthetized in megakaryocytes or platelets, rather it is taken up from the blood plasma and stored in α-granules, although the exact endocytic pathway is not fully understood. Interestingly, on the cell surface of megakaryocytes, galectin-8 directly binds FV in a carbohydrate-dependent manner, and this interaction is most likely mediated by several N-linked glycans located on FV (218). In line with this result, genetic or pharmacological blockade of galectin-8 function could strongly inhibit FV uptake. Thrombopoietin (TPO) treatment in megakaryocytes could inhibit galectin-8 expression on the cell surface, and consequently, FV uptake was also inhibited, indicating that this molecular mechanism only exists in premature megakaryocytes (218).

Galectin-8 exposure was observed upon activation of vascular cells, suggesting that galectin-8 could potentially link both processes of platelet activation and inflammation on the surface of endothelial cells. vWF is stored in the Weibel-Palade bodies of endothelial cells and exposed on the cell surface under thrombotic and inflammatory conditions, responsible for linking platelet GPIb-GPV-GPIX complex to the activated endothelium (181, 219). Galectin-8 can activate endothelial cells, thereby increasing vWF secretion and enhancing platelet adhesion. Interestingly, both galectin-1 and galectin-3 can bind vWF in endothelial cells, thereby modulating vWF-mediated thrombus formation (181). Furthermore, galectin-8 increases the expression of pro-inflammatory molecules, including chemokines and interleukins, thereby mediating the interactions among leukocytes, blood platelets, and inflamed vascular endothelium. Galectin-8 enhanced the release of inflammatory cytokines and chemokines, namely CCL2, CXCL3, CXCL8, CXCL1, GM-CSF, IL-6, and CCL5, thereby influencing angiogenic response and recruitment of immune cells (130).

#### 4.2.7. Galectin-9

Inflammatory mediators can modulate the expression of galectin-9. Phorbol 12-myristate 13-acetate (PMA), IFN-γ and LPS can upregulate galectin-9 levels, indicating an important role of galectin-9 in inflammatory diseases (220–222). Elevated blood galectin-9 levels were found in patients with stroke after 6 days of ischemic insult (223). In the rat model of ischemic stroke, galectin-9 expression is also increased, indicating that blocking galectin-9 function may have protective effects in stroke (38). Increased serum galectin-9 levels were also observed in patients with stable coronary artery disease, suggesting an important role of galectin-9 in cardiovascular pathology (224).

Disruption of the blood–brain barrier is responsible for the development of hemorrhagic and thromboinflammatory diseases, including stroke and cerebral malaria (173, 225). Interactions between galectin-9 and CD146 facilitate the sequestration of infected red blood cells and pro-inflammatory lymphocytes in the vasculature of the central nervous system, thereby promoting the disruption of the blood–brain barrier (226). Galectin-9 also acts as an astrocyte-microglia communication signal by potentiating the release of TNFα from microglial cells (227). Endothelial cells are also major sources of galectin-9, which is exposed on the cell surface upon inflammation and endothelial cell activation (228). Therefore, galectin-9 has been proposed as a mediator of thromboinflammation by inducing the capture of immune cells and blood platelets to the activated endothelium. Galectin-9 was recently discovered as a novel platelet agonist that induced platelet adhesion, spreading, aggregation and degranulation through interactions with platelet hem immunoreceptor tyrosine-based activation motif (ITAM) receptors GPVI and C-type lectin-like receptor 2 (CLEC2), suggesting that galectin-9 regulates thrombus formation (229). Although lactose-dependent blockade of galectin-9 function was observed in platelet aggregation, and this suggests a carbohydrate-dependent action on (hem) ITAM signaling in platelets (229), further investigation is necessary to identify the exact molecular mechanisms of how galectin-9 may bind (hem) ITAM receptors.

Thrombotic complications are frequently observed in severe acute respiratory syndrome coronavirus 2 (SARS-CoV-2) syndrome patients. A recent study reported a strong elevation of blood galectin-9 levels in patients with SARS-CoV-2 infection, suggesting a modulatory effect of galectin-9 in platelets, immune cells, and endothelium (230), which may accelerate thromboinflammation in the infected lung tissues. Galectin-9 levels were higher in the plasma of hospitalized SARS-CoV-2 patients who died, compared to survivors (230). Although these studies highlighted galectin-9 function in thromboinflammation, the exact molecular mechanism, regulated by galectin-9 in infected patients awaits further investigations.

During atherosclerosis, galectin-9 modulates inflammation through the activation of T cells, monocytes, and macrophages (231). The imbalanced immune functions mediated by Th1 and Th2 cells are important hallmarks of the pathogenesis of atherosclerosis. Th1-induced immune responses enhance the development of atherosclerosis, while Th2-mediated responses attenuate the atherosclerosis-enhancing effects of Th1 cells and provide protection (232). Immunologic protective effects in atherosclerosis are also contributed by other subsets of CD4+ T and Treg cells. Although galectin-9 induces apoptosis in CD4+ Th1 cells, on the other hand, galectin-9 can activate T cells, thereby expanding Th1 cells and central memory cells. Furthermore, galectin-9 induces the differentiation of naïve T cells to Treg cells. Consistently, the numbers of Treg cells are reduced in *Lgals9^−/−^* mice (233). The blockade of galectin-9-Tim-3 interactions increases macrophage infiltration in atherosclerotic plaques (231). In an experimental model of preeclampsia, characterized by abnormal macrophage polarization, decreased Tim-3 levels were also correlated with the pathogenesis of the disease (234). Interestingly, galectin-9 treatment reversed inflammation-associated damages by activating and upregulating Tim-3 expression in immunosuppressive macrophages (234). Besides, the expression of human galectin-9 in transgenic mice also induced differentiation and activation of immunosuppressive CD11b1-Ly-6G-positive granulocytes (235). Recent studies by Zhu et al. demonstrated that the serum level of galectin-9 was reduced significantly in patients with coronary artery disease (224). Application of exogenous galectin-9 increased the number of Tregs cells and inhibited Th17 cell function, thereby increasing the production of TGFβ and decreasing the synthesis of IL-17 (236, 237). Patients with the acute coronary syndrome also displayed decreased mRNA levels of galectin-9, TIM-3, and forkhead box P3 (Foxp3) in peripheral blood mononuclear cells (238). Altogether these data indicate that upregulation of the galectin-9-Tim-3 signaling pathway attenuates atherosclerotic plaque development by inhibiting the recruitment of monocytes/macrophages and effector T cells and by enhancing the expansion of Tregs. Under flow conditions, galectin-9 promotes the adhesion of B cells to the vascular endothelium by decelerating their transendothelial migration (239). Galectin-9 also acts as an eosinophil and monocyte chemoattractant, inducing cell activation, differentiation, degranulation, and ROS production (240–242). Galectin-9 also mediates neutrophil chemotaxis, thereby inducing neutrophil activation and migration (243, 244).

Stimulation of monocytic cells with LPS induced nuclear translocation of galectin-9. Only intracellular galectin-9 was shown to regulate promoter activity of pro-inflammatory genes such as IL-1α, IL-1β, and IFNγ (245). Furthermore, intracellular galectin-9 could activate transcription factors such as nuclear factor (NF)-IL-6 and AP-1 but did not activate the NF-κB pathway (245). Interestingly, extracellular galectin-9 does not stimulate these processes (245).

#### 4.2.8. Galectin-10

Eosinophilic granulomatosis with polyangiitis (EGPA) is a rare chronic inflammatory systemic disease that occurs in patients with bronchial asthma and is associated with significant blood and tissue eosinophilia (246). Galectin-10 functions have been studied primarily in the context of this disease. Eosinophils of patients with EGPA are primed to generate cytolytic eosinophil extracellular traps (247). This process was shown to rely on the production of ROS and histone-citrullination resulting in the release of free galectin-10 and membrane-bound intact eosinophil granules, thereby increasing serum IL-10 levels (247). In ruptured atherosclerotic plaques and arterial thrombi, eosinophil extracellular trap formation was also detected (248, 249). Whether galectin-10 may induce extracellular trap formation of eosinophils or other immune cells in a pro-thrombotic environment is still unknown.

#### 4.2.9. Galectin-12

Galectin-12 is highly expressed in adipose tissues and regulates the differentiation and expansion of preadipocytes (250). *Lgals12^−/−^* mice have increased insulin sensitivity and glucose tolerance, contributing galectin-12 function to diabetes and metabolic syndrome, and indicating the potential involvement in cardiovascular diseases (251). Ablation of galectin-12 decreased the secretion of MCP-1, TNF-α, and IL-6 in LPS- and saturated fatty-acid-stimulated macrophages, suggesting the involvement in the pathogenesis of atherosclerosis (156). The ablation of galectin-12 also results in the polarization of macrophages into the M2 subset. The negative regulation of M2 macrophage polarization by galectin-12 causes enhanced inflammation and decreases insulin sensitivity in adipocytes (252). Galectin-12 deficiency in mice leads to M2 macrophage polarization that consequently reduced foam cell formation and pro-inflammatory cytokine production thereby reducing atheroma formation in an atherosclerosis animal model (156). Based on these results, it was proposed that inhibition of galectin-12 may be a novel therapeutic strategy to slow down the progression of atherosclerosis.

#### 4.2.10. Galectin-13

High levels of galectin-13 are detected in the blood of pregnant women at the early stage of pregnancy. In the placenta, galectin-13 is expressed in syncytiotrophoblasts and blood vessels. Reduced levels of galectin-13 caused by genetic mutations are associated with a high risk of preeclampsia. In parallel to its decreased placental expression, an enhanced galectin-13 shedding from the cell surface was shown to contribute to the elevated levels of serum galectin-13 during the third trimester of pregnancy in women with preterm preeclampsia. The release of galectin-13-positive microvesicles was proposed to aggravate placental ischemic stress during preeclampsia. Preeclampsia and hypertension during pregnancy are associated with an elevated risk of arterial thrombosis. The similar pathological observation was described in the blood of rare liver and blood clotting disorder (HELLP syndrome).

#### 4.2.11. Galectin-14

The dysregulation of galectin-14 is also involved in hypertension and preeclampsia (253). However, the studies focusing on galectin-14-mediated regulatory mechanisms are limited.

### 4.3. Galectin-mediated crosstalk between cancer and thrombosis

Thrombotic complications, such as a deep vein or arterial thrombosis and pulmonary embolism are frequent pathogenic events in patients with cancer (171, 254). Elevated platelet count and a pro-coagulant tumor microenvironment are indicators of a poor prognosis, implying a high risk of thromboembolic events and resistance to anti-cancer therapies (171). In mouse models of melanoma, breast, and colon cancer, platelets interact with circulating tumor cells, thereby protecting them from shear stress and NK-cell-induced destruction and enhancing tumor metastasis (255, 256). Platelets also induce EMT and recruitment of granulocytes to the metastatic niches (171, 172, 257). Platelet membrane receptors, integrins, and glycoproteins actively contribute to complex interactions between blood platelets, tumor cells, vascular endothelium, and fibrin clots, thereby promoting tumor growth and metastasis (171, 200, 257, 258). Tumor cells can directly trigger platelet activation through binding to the platelet-resident receptors, thereby amplifying pro-thrombotic and pro-coagulant phenotypes (171). Cancer cells can also trigger indirect platelet activation by inducing the release of tissue factors and ECM proteins from activated vascular endothelium, which create an active surface for platelet adhesion and thrombosis (171).

Abnormal O- and N-glycosylation and glycan binding, triggered by the tumor microenvironment, could potentially modify galectin-mediated responses, thereby influencing thrombotic and hemostatic events in cancer patients (170, 259, 260). Recently, we showed that platelet GPVI interacts with a tumor-resident galectin-3 through the binding to its collagen-like domain, expressed on the surface of breast and colon cancer cells (200). This interaction could induce platelet activation and granule release, thereby enhancing tumor cell extravasation and lung metastasis (200). More recently, galectin-3 has also been proposed as a ligand of platelet GPVI on ovarian cancer cells (261).

**Figure 4:**
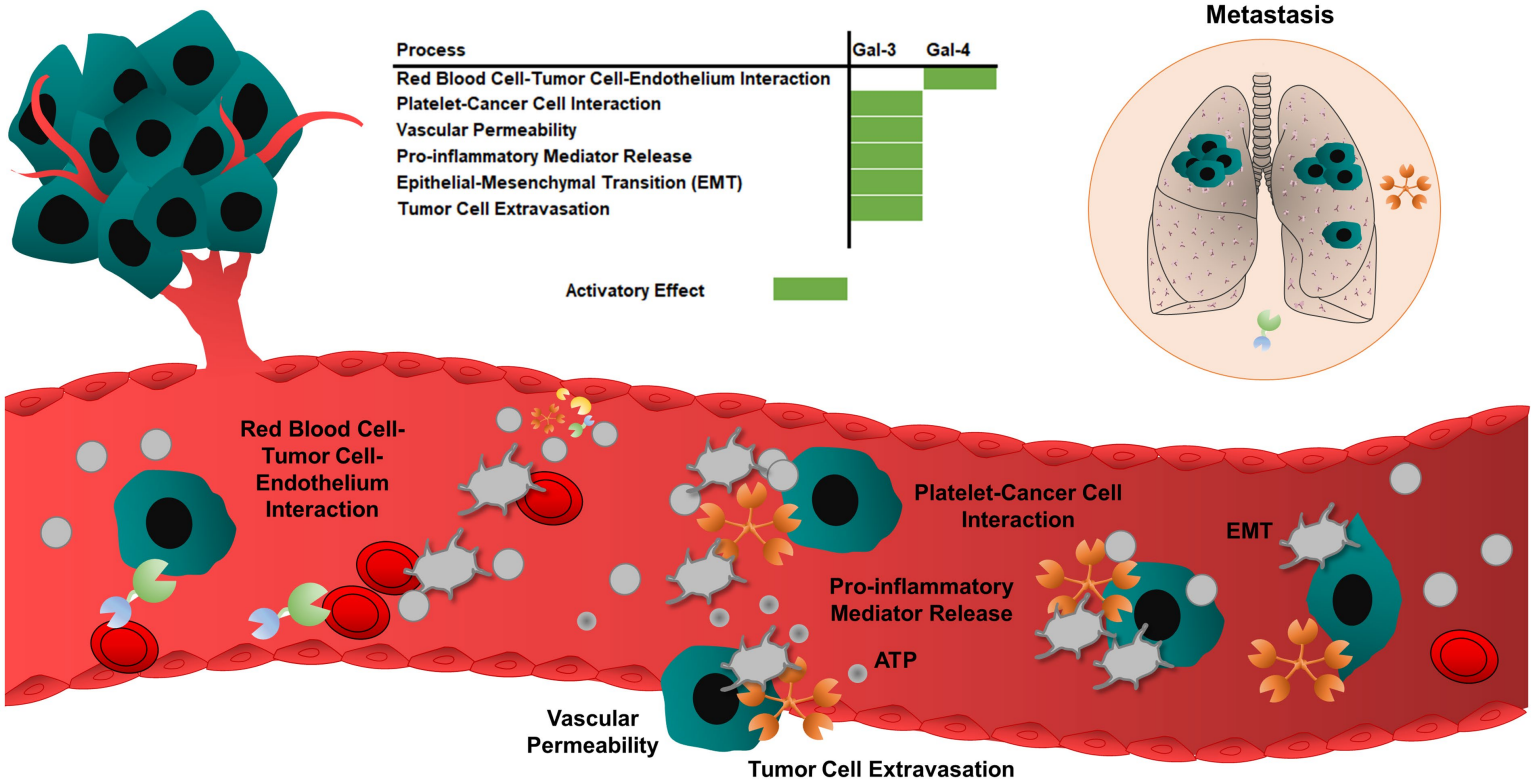
Invasive cancer cells enter into the blood circulation where they can build physical and functional interactions with platelets, red blood cells, and vascular endothelium. These interactions support the metastatic spread of cancer cells, thereby inducing their survival and proliferation at distant organs. Hematogenous metastasis is strongly influenced by tumor-resident galectins (galectin-3 and galectin-4) that interact with glycoproteins on the surface of platelets and red blood cells. Galectin-3 promotes platelet adhesion and activation through binding to platelet GPVI, thereby enhancing tumor cell extravasation and subsequent metastasis. These interactions also contribute to the epithelial-mesenchymal transition and pro-inflammatory phenotype, further sustaining the invasive and metastatic potential of cancer cells.

During thrombotic events, red blood cells enhance platelet interactions with thrombi by increasing the viscosity of the blood and margination of platelets toward endothelium. Galectin-4 can form a bridge between cancer cells, vascular endothelium, and red blood cells. The CRD domain of galectin-4 interacts with human blood group antigens (ABH) (262). The tumor cell-resident galectin-4 possibly mediates metastasis through interactions with ABH antigen-binding glycoproteins present on the surface of red blood cells (263) (Figure 4).

## 5. Clinical application of galectin inhibitors in cancer and thromboinflammation

Galectins have multiple effects on cancer and many cell types involved in thromboinflammation, therefore therapeutic strategies have been applied to target galectin-mediated signaling in immune and cancer cells. However, the functional blockade of platelet-resident galectins has been out of focus in these studies. During the last years, several blocking antibodies, peptides, competitive carbohydrates, and small non-carbohydrates have been developed to selectively inhibit galectin isoforms. Some of the galectin blockers are currently in clinical studies to inhibit certain pathogenic functions in patients with solid cancers. In this chapter, we summarize the results of preclinical studies using different mouse models (Table 1), and we also discuss important clinical trials.

**Table 1:** Preclinical and clinical applications of galectin inhibitors in thromboinflammation and cancer.

Inhibitor	Galectin	Cancer type	Effect	Refs
Anginex & analogues (6DBF7, DB16, DB21)	1	Teratocarcinoma	*In vivo:* Inhibition of angiogenesis and tumor growth	(55, 264–266)
F8.G7 (8F4F8G7)	1	Epstein–Barr-Virus (−associated PTLD) Kaposi’s sarcoma	*In vitro:* Inhibition of T-cell apoptosis; *in vivo:* dose-dependent delay of tumor growth and angiogenesis	(267, 268)
OTX008/PTX008/0018	1	B-cell precursor acute lymphoblastic leukemia; breast, colon, head and neck, ovarian and prostate cancer; hepatocellular carcinoma; melanoma; non-small lung carcinoma ovarian cancer *clinical phase I trial* in patients with solid tumors	*In vitro:* Inhibition of tumor cell aggregation, adhesion, migration, invasion and proliferation; *in vivo* and *in vitro:* inhibition of angiogenesis and tumor growth, normalization of tumor vasculature, additive or synergic effects with chemotherapeutic drugs	(225, 269–276)
Thiogalactoside (TDG)	1	Melanoma, breast and colon cancer	*In vivo:* Dose-dependent suppression of tumor growth and angiogenesis; increased CD8^+^ and CD4^+^ T-cell tumor infiltration; reduction of lung metastasis	(277–279)
Galectin-2 blocking-antibody	2	Breast cancer	*In vitro* and *in vivo*: Arrested tumor cell growth and reversed immunosuppressive phenotype of M2 macrophages	(280)
GM-CT-01 (Davanat)	1,3	Melanoma, solid tumors (colorectal, breast, lung, prostate, head and neck)	*Preclinical studies & clinical phase 1/2 trial*: Increased cytotoxicity of CD8+ TIL and their IFN-γ secretion in a dose-dependent manner, correct TIL anergy and efficient and long-lasting anti-tumoral immune response following peptide vaccination	(281–284)
GR-MD-02 (Belapectin)	1,3	Melanoma, head and neck cancer	*Clinical phase Ib trial*: Positive response and disease control rate in combination with KEYTRUDA® (pembrolizumab) in metastatic melanoma and head and neck cancer patients	(285)
Synthetic lactulose amines (SLA)	1,3	Melanoma, small cell lung carcinoma	*In vitro:* Induction of tumor cell apoptosis, inhibition of endothelial cell morphogenesis and tumor cell aggregation in the presence of protein 90 K	(286)
TDG ester derivatives	1,3,7,9N	Non-small lung carcinoma, hormone-refractory prostate cancer	*In vitro:* Inhibition of tumor cell migration	(287)
G3-C12	3	Breast cancer, prostate cancer	*In vitro*: Inhibition of lung metastasis and improved anti-cancer activity in combined therapy with N-(2-hydroxypropyl) methacrylamide (HPMA) compound and 5-FU	(288, 289)
Galectin-3C	3	Ovarian cancer, multiple myeloma	*In vitro:* Reduced proliferation, migration, invasion and angiogenic potential of cancer cells. Increased anticancer activity of bortezomib in human multiple myeloma cells	(290)
TFD10	3	Prostate cancer	*In vitro:* Inhibition of galectin-3-mediated angiogenesis, tumor cell-endothelial cell interactions, and impaired apoptosis of activated T cells	(291)
Anti-GPVI blockers	3	Breast cancer, coloncancer and ovarian cancer	*In vitro*, *in vivo* and *ex-vivo*: Inhibition of galectin-3-GPVI-mediated platelet-cancer cell interactions and attenuation of invasive potential of cancer cells	(200, 261, 343)
RN1	3	Pancreatic cancer	*In vivo and vitro:* inhibition of tumor cell growth of patient-derived xenografts	(292)
GB1211	3	Non-small lung carcinoma	*Clinical phase 1/2 trial:* Investigation of the Safety and Efficacy of GB1211 (a Galectin-3 Inhibitor) in Combination With Atezolizumab in Patients With Non-Small Cell Lung Cancer (NSCLC)	(293, 294)
GCS-100	3	Multiple myeloma, acute myeloid leukemia	*In vitro:* Inhibition of myeloma cell growth by induction of apoptosis	(295–297)
TD139	3	Thyroid cancer	*In vitro:* Suppression of anoikis resistance and cell motility in thyroid cancer cells	(298, 299)
GB1107	3	Lung carcinoma	*In vitro:* Potentiation of PD-L1 immune checkpoint inhibitor to increase T-cell infiltration	(300)
Modified Citrus Pectins (MCP)	3	Ovarian cancer	*In vitr*o: Synergic effect with chemotherapeutic drugs to kill cancer cells and reduction of cancer cell adhesion, migration and invasion	(301–303)
D-galactal-benzimidazole hybrid	8	Breast cancer	*In vitro:* Dose-dependent reduction of proinflammatory cytokines (IL-6 and -8)	(304)
Recombinant Galectin-9	9	Chronic myeloid leukemia	*In vitro:* Sensitizing of cancer cells to tyrosine kinase inhibitor treatment through activation of proapoptotic Noxa pathway	(305)
TSR-022, LY3321367 and MBG453 (humanized anti-Tim-3 antibodies)	9	Solid tumors	*Clinical phase 1/2 trials:* Inhibition of Tim-3/galectin-9 interaction prevents T cell inhibition and enhances anti-tumor immunity in combination with anti-PD1 and -PDL1 therapy	(306–310)
LYT-200	9	Solid metastatic tumors	*Clinical phase 1/2 trial:* Inhibition of Gal-9 and PD-1 interaction; removal of immunosuppressive effect on effector T-cells	(131)

### 5.1. Galectin-1

Thiodigalactoside (TDG) is a synthetic disaccharide with a high affinity for galectin-1, thereby blocking galectin-1-mediated tumor angiogenesis and immune response, and preventing oxidative stress in a non-selective manner. In mouse models of breast and colon cancers, TDG reduced the number of CD31-positive endothelial cells, vessel formation, and the number and size of lung metastases. TDG could also prevent galectin-1 binding to CD44 and CD326 receptors on the surface of cancer stem cells (277, 278, 311). Although it was considered that TDG is a non-selective blocker for galectin-1, TDG treatment did not induce any additional tumor-suppressive effects in *Lgals1^−/−^* mice (311).

Anginex (β Pep-25) is an anti-angiogenic peptide, which contains anti-angiogenic factor motifs to bind the CRD domain of galectin-1. Anginex also inhibits galectin-1 uptake in endothelial cells, thereby inhibiting Raf/MEK/ERK kinase signaling (264). Anginex rendered tumor-associated endothelial cells sensitive to radiotherapy and displayed a synergistic effect with a suboptimal dose of carboplatin, triggering radiation-induced tumor regression (312). Nanoparticles of Anginex were used in a galectin-1-overexpressing breast cancer mouse model, showing a robust reduction in tumor growth, and similar results were obtained with Anginex analogues (6DBF7, DB16, DB21) (265). However, Anginex treatment has limitations, as reduced stability and half-life were observed with limited effects on endothelial cells to radiotherapy in neovascularized tumors, but not on cancer cells. Anginex also interacts with other galectins, such as galectin-2, 7, 8 N, and 9 N, possibly blocking their biological activities as well (266).

Galectin-1-VEGFR-2 interaction induces tumor outgrowth and limits the efficacy of anti-VEGF therapy on the endothelium (313). Therefore, a monoclonal anti-galectin-1 antibody, F8.G7, was designed to target galectin-1-associated VEGF signaling (267, 314). Kaposi sarcoma treatment with F8.G7 antibody in mice strongly inhibited tumor growth, angiogenesis, and VEGFR2-induced signaling pathway (267). F8.G7 treatment could also increase anti-tumor immunity by increasing the infiltration of T lymphocytes, and the release of IFNγ and IL-17 (267). Although non-canonical VEGF signaling was targeted by F8.G7 treatment, the canonical VEGF signaling still induced tumor angiogenesis, limiting the effectiveness of this treatment.

OTX008 is a calixarene derivative designed to target galectin-1 at a distant site of CRD, compared to Anginex (269). OTX008 could inhibit the invasion of laryngeal squamous cell carcinoma and ovarian endometrioid adenocarcinoma cells by inhibiting ERK1/2 and AKT-dependent cell survival signaling, CDK1-mediated G2/M cell cycle arrest and angiogenesis (270, 315). OTX008 could inhibit tumor growth with a strong reduction of galectin-1, Ki-67, and VEGFR-2 expressions *in vivo* (270). OTX008 has synergistic effects with immuno- and chemotherapeutic agents, such as everolimus, regorafenib, sunitinib, 5-FU, docetaxel, oxaliplatin, and cisplatin (316). The potential effects of OTX008 on thrombosis and hemostasis have not been investigated; therefore, we only speculate that OTX008 may represent a potential therapeutic approach to target galectin-1-mediated platelet adhesion and platelet–neutrophil interactions in cancer. However, these protective effects appear to depend on the pathogenesis of the thrombotic disease as an opposite therapeutic effect of galectin-1 treatment was observed in a mouse model of acute myocardial infarction (184). Treated mice with recombinant galectin-1 could improve cardiac homeostasis and post-infarction remodeling by attenuating tissue damage and preventing cardiac inflammation (184).

### 5.2. Galectin-2

Targeting galectin-2 function in triple-negative breast cancer was proposed to be effective in reverting immunosuppression induced by M2-like macrophages (280). Injection of 4 T1 cells into the mouse mammary fat pad, followed by injection of therapeutic antibody against galectin-2 function, arrested tumor growth and successfully reversed the immunosuppressive phenotype (280). Although validated results are limited, it was proposed that blocking galectin-2 with selective antibodies or nanobody clones would be an excellent strategy to target galectin-2-mediated collateral arteriogenesis, myocardial infarction, immune cell adhesion, inflammation, and T-cell apoptosis in atherosclerosis (317). Therefore, it would be necessary to investigate further whether galectin-2 blockade may inhibit several routes of galectin-2-mediated malignancy, including tumor growth, immunosuppression, and thromboinflammation.

### 5.3. Galectin-3

Several galectin-3 inhibitors have been developed and tested in preclinical mouse models. RN1 is a polysaccharide isolated from flowers of *Panax pseudo-ginsieng*, which binds and downregulates galectin-3-associated signaling pathways, thereby reducing the proliferation rate of pancreatic cancer cells and the growth of patient-derived xenografts both *in vitro* and *in vivo* conditions (292).

TFD10 is a glycopeptide isolated from polar cod fish that displayed inhibitory effects in prostate cancer metastasis by blocking galectin-3-mediated angiogenesis and tumor cell-endothelial cell interactions and inhibiting the apoptosis of activated T cells (291).

A dominant negative form of galectin-3 (Galectin-3C) was developed to block endogenous galectin-3 functions (290). Galectin-3C strongly reduced the proliferation, migration, invasion and angiogenic potential of human and mouse ovarian cancer cells (290). Galectin-3C could increase the anticancer activity of bortezomib in human multiple myeloma cells (290).

G3-C12 oligopeptide is a frequently used galectin-3-CRD domain blocker, which inhibited lung metastasis in a mouse model of breast cancer (288). The protective effects of G3-C12 were improved in combined therapy with N-(2-hydroxypropyl) methacrylamide (HPMA) compound and 5-FU. G3-G12-HPMA complex strongly improved the anti-tumor activity of 5-FU in nude mice bearing tumor of human PC-3 cancer cells (289).

GM-CT-01 (DAVANAT) is a modified vegetal galactomannan oligomer that strongly binds the dimer interface of galectin-1 and galectin-3 (318). GM-CT-01 enhances the infiltration of T cells into the tumor microenvironment and induces IFNγ synthesis (319). GM-CT-01 was administered in combination with 5-FU in patients with metastatic solid cancers that failed initial therapy (NCT00388700). In clinical trial phase II, 70% of the patients were stabilized at the highest DAVANAT dose level, 46% of patients experienced an increase in life expectancy, and merely 41% of patients developed serious adverse effects, compared to the best standard of care. Food Drug Administration (FDA) approval was given to progress to clinical trial phase III studies, although this was not followed up as of now (320). Multiple factors led to the withdrawal of two further clinical trial phase II studies using GM-CT-01 therapy (NCT00388700 and NCT00386516). The vaccine form of GM-CT-01 is currently in clinical trial phase II, injecting patients with diffuse melanoma (NCT01723813).

GR-MD-02 (Belapectin) binds galectin-3 and preclinical settings could improve liver function by reducing collagen deposition, fibrosis in non-alcoholic steatohepatitis (NASH), and cirrhosis (321). GR-MD-02 increased the efficacy of immunotherapeutic agents such as the checkpoint inhibitor pembrolizumab (322). Recently, GR-MD-02 in combination with ipilimumab or pembrolizumab is being investigated in melanoma, non-small cell lung cancer, and squamous cell head and neck cancer in a clinical trial phase I without published results and still ongoing studies (NCT02575404 and NCT02117362).

Modified citrus pectin (MCP) is originally a β-galactoside nutritional supplement, which inhibits galectin-3 function through multiple mechanisms. Indeed, MCP could inhibit cell adhesion in galectin-3-overexpressing tumor cells, reducing tumor cell aggregation and adhesion to the endothelial cells, thereby inhibiting the metastatic potential of cancer cells (323, 324). In prostate cancer cells, MCP enhanced anoikis by inhibiting cyclin B and cdc function, which subsequently induced G2/M cell cycle blockade and apoptosis (325). Furthermore, MCP function was also studied in cardiovascular and renal diseases. MCP could inhibit aldosterone-induced cardiac and renal fibrosis and improve cardio-renal dysfunction (326). MCP could prevent fibrosis in rodents with heart failure and hyperaldosteronism and inhibit obesity-associated adipose tissue inflammation and differentiation of adipocytes and atherosclerotic plaque progression (327–329). Additionally, MCP reduced myocardial injury after ischemic reperfusion (330).

A derivative of MCP, PectaSol-MCP was evaluated in clinical trial phase II (NCT01681823) regarding its capability to improve prostate-specific antigen (PSA) kinetics in men with increased PSA levels and biochemically relapsed prostate cancer, PSA progression was inhibited in 58% of patients. PSA doubling time, a prognostic marker predicting the development of metastasis, lengthened in 75% of patients versus baseline indicating a reduced risk of metastatic spread (331). However, the major limitation of this study was the lack of a placebo arm, which, given the perceived positivity of PectaSol-MCP, was not conducted.

GCS-100 is an MCP-derived polysaccharide and was used to study galectin-3-mediated pro-tumorigenic effects. GCS-100 could induce apoptosis in multiple myeloma cells, which displayed strong resistance to doxorubicin, melafalan, bortezomib, and dexamethasone (295). GCS-100 treatment together with a BH3-mimetic also induced apoptosis in acute myeloma cells and interestingly these effects were associated with the induction of P53 (296). Although GCS-100 was well tolerated by patients in clinical trial phase II, partial response and stable disease were observed in patients with recurrent chronic lymphocytic leukemia (NCT00514696).

TD139 binds the CRD domain of galectin-3 with a strong anti-inflammatory and anti-fibrotic potential in mouse models of lung injury and fibrosis (332, 333). TD139 downregulates galectin-3 expression on broncho-alveolar lavage macrophages inhibits fibroblast activation and reduces the levels of plasma biomarkers associated with idiopathic fibrosis progression (334, 335). TD139 treatment also inhibits pro-tumorigenic effects in thyroid cancer cells by attenuating AKT phosphorylation and reducing the expression of MMP2 and β-catenin, which leads to the suppression of anoikis resistance, motility and invasion (336). The β-glycan receptor dectin-1 is a potential biomarker of lung fibrosis (337). Dectin-1 is highly expressed in bone-marrow-derived myeloid cells, including monocytes, macrophages, and neutrophils (338). Interaction between dectin-1 and galectin-3 is required for pathogen recognition and TNF-α response in bone marrow-derived myeloid cells (339). It was proposed that this interaction is involved in platelet aggregation and thrombosis, thereby enhancing atherothrombosis and myocardial infarction (201). Plasma galectin-3 levels are increased in patients with coronary artery diseases and atherosclerosis (201, 340). In *ApoE^−/−^* atherosclerotic mice, elevated plasma galectin-3 levels were detected and TD139 could effectively inhibit galectin-3-mediated platelet activation and thrombosis (201). Furthermore, TD139 also inhibited microvascular thrombosis and prevented myocardial infarction. Therefore, TD139 was proposed as a potent galectin-3 inhibitor improving atherothrombosis and myocardial infarction (201).

Interestingly, the collagen-like domain of galectin-3 binds GPVI on the platelet surface, thereby inducing platelet activation and adhesion (200, 261). Blockade of GPVI was proposed as an alternative therapeutic approach, which inhibits galectin-3-mediated platelet signaling and platelet-mediated pro-tumorigenic effects (171). Recently, we showed that functional inhibition of GPVI with F (ab′)2 fragments of Jaq1 antibody could increase the intratumoral efficacy of chemotherapeutic drugs (341). Jaq1 F(ab′)2 antibody also blocked platelet adhesion to galectin-3-positive cancer cells, thereby inhibiting platelet-tumor cell interaction and consequent tumor metastasis (200). Soluble dimeric GPVI - Revacept is a clinically relevant competitive inhibitor of GPVI, which directly binds collagen thereby removing collagen fibers from the platelet surface (171, 342). Similar to the Jaq1 F(ab′)2 antibody, Revacept and other soluble dimeric-like GPVI blockers also inhibited galectin-3-mediated platelet-cancer cell interaction and lung metastasis (200, 261). Using galectin-3-positive HT29 human colon cancer cells, Dovizio et al. showed that Revacept had an inhibitory effect on COX-2 and platelet-mediated EMT *in vitro* (343). In ovarian cancer, galectin-3 is a ligand of platelet GPVI, and this interaction regulates platelet-dependent tumor cell extravasation and pro-inflammatory signature in cancer cells (261). In human ovarian cancer on a chip model, Revacept treatment also inhibited galectin-3-GPVI-mediated platelet-cancer cell interactions and pro-inflammatory phenotype, thereby attenuating the invasive potential of cancer cells (261).

### 5.4. Galectin-4

Intracellular galectin-4 interacts with β-catenin, leading to the inhibition of the Wnt pathway-associated cell proliferation and cancer growth (102). Galectin-4 is also released to extracellular space and high galectin-4 levels in the blood were observed in patients with metastatic colon, hepatocellular, and breast cancers (108, 344–346). Extracellular galectin-4 inhibited the secretion of IL-17 from activated T cells in the colon, thereby inducing apoptosis and preventing inflammation (347). Interestingly, extracellular galectin-4 could induce apoptosis in galectin-4-deficient colorectal cancer cells, while in galectin-4-positive colorectal cancer cells, inflammatory chemokine gene expression and secretion were downregulated (348). In line with this result, an anti-galectin-4 antibody that inhibits extracellular galectin-4 function could increase cell proliferation with the concomitant secretion of chemokines (348). In other studies, lower expression of galectin-4 was associated with an advanced form of colorectal cancer, and galectin-4 overexpression could improve the efficacy of camptothecin treatment (105, 345). Galectin-4 binds carbohydrate groups on red blood cell group antigens and cholesterol 3-sulfate and sulfatides within lipid raft membranes through its CRD domains (263, 349). Further studies are required to design blocking approaches to interfere with these binding sites.

### 5.5. Galectin-7

Based on the crystal structure of galectin-7A, 2-O-galactoside benzyl-phosphorane was synthesized which displayed a 60-fold increased affinity for this galectin isoform, as compared to galactoside (350). Recombinant human galectin-7 protein could inhibit apoptosis in Jurkat T cells, indicating on immunosuppressive function (351). This proapoptotic effect was inhibited by targeting the dimer interface of galectin-7, thereby disrupting homodimerization (352). Galectin-7 also regulates immune response to transplantation by promoting Th1/Th2 differentiation toward Th1, which results in increased rejection of grafted hearts (216, 353). Recently, gene therapy was developed based on galectin-7-siRNA knock-down with ultrasound-targeted microbubble destruction that could prevent acute rejection during allograft heart transplantation (353).

### 5.6. Galectin-8

The inhibition of one CRD domain of galectin-8 was sufficient to inhibit the biological function of this isoform. D-galactal-benzimidazole hybrid was designed to block galectin-8 function through binding to its N-terminal domain (304). Although the D-galactal-benzimidazole hybrid showed no effect on the proliferation of human breast cancer cells, the secretion of pro-inflammatory cytokines, IL-6 and IL-8, was reduced in a dose-dependent manner (304). The CRD of galectin-8 and the dominant negative version of galectin-8 (Gal-8 N) were both blocked by 3′-sialyl lactose in a mouse model of corneal allogeneic transplantation, resulting in severely inhibited angiogenesis (351).

### 5.7. Galectin-9

Galectin-9 inhibition in hepatocellular carcinoma cells showed increased cell proliferation and migration *in vitro* (354). Patients with higher galectin-9 levels had better survival than those who were negative for galectin-9 expression (135). Adding recombinant galectin-9 into cell culture promoted cell death in colorectal carcinoma with KRAS mutation (355). This therapeutic approach could also potentiate rapid endocytosis and accumulation of rsGal-9 in lysosomes, resulting in lysosome swelling and accumulation of autophagosomes and cell death (356). Treatment of chronic myeloid leukemia cells with human galectin-9 rendered the cells sensitive to tyrosine kinase inhibitors, thereby inducing apoptosis through modulation of activating transcription factor 3-Noxa proapoptotic pathway (305).

Galectin-9 regulates anti-tumor immunity through modulating Tim-3/galectin-9, PD-1/PD-L1, and dectin-associated immune checkpoints. Interestingly, Galectin-9 binds PD-1 which is distinct from the PD-L1-binding site (131). Anti-Tim-3 antibodies have been generated to block the interactions with galectin-9 and also other ligands such as CEA cell adhesion molecule 1 (CEACAM1) and high-mobility group protein B1 (HMGB1) (306). These antibodies could strongly prevent T cell inhibition and enhance anti-tumor immunity. A humanized anti-Tim-3 antibody, TSR-022 has been evaluated in clinical trial phases I and II and resulted in stable diseases in patients with progressed solid tumors (307). TRS-022 therapy was also combined with an anti-PD1 antibody (TSR-042) (307). The first results showed good tolerance of TRS-022/TSR-042 in melanoma and non-small cell lung cancer (NSCLC) (308). Another anti-Tim-3 antibody, LY3321367 also presented a safety profile and modest anti-tumor activity or in combination with LY300054, an anti-PD-L1 antibody (309). Similar combinations were also developed using MBG453, anti-Tim-3 antibody and spartalizumab, an anti-PD1 antibody, which presented better outcome and antitumor activity as compared to monotherapy (310).

LYT-200 is an IgG4 monoclonal antibody, designed to inhibit the activity of galectin-9. It was given orphan drug designation by the FDA and it is tested in an open-label, uncontrolled, multicenter phase 1/2 study with a dose escalation phase and a cohort expansion phase in patients with relapsed/refractory metastatic solid tumors, including PDAC, colorectal cancer, and cholangiocarcinoma (NCT04666688). The study was posted in 2020 and is currently in its recruiting stage. Galectin-9-CD146 interaction contributes to cerebral malaria, inducing the adhesion of red blood cells, and T cells, and the rupture of the blood–brain barrier (226). Blockade of CD146 counter receptor using AA498 could block galectin-9-mediated cell adhesion and inflammation and disease pathogenesis in cerebral malaria (226). CD146 can also bind to galectin-1 and galectin-3 (226). Whether such an approach interferes with interactions of other galectins requires further investigation.

Galectin-9 can also support platelet adhesion and activation through interactions with hemi-ITAM receptors GPVI and CLEC2 (229). *In vitro* blockade of galectin-9-mediated platelet adhesion using an anti-GPVI Jaq1 Fab fragment and anti-CLEC2 AYP1 antibody could inhibit galectin-9-mediated platelet adhesion and activation (229). Similar inhibition was observed using a pan galectin inhibitor, lactose (229). Thrombosis occurs as a consequence of endothelial disruption or rupture of atherosclerotic plaques (174). One would expect beneficial effects of galectin-9 inhibition in atherosclerosis. However, the blockade of galectin-9 function was shown to increase atherosclerotic plaque formation (231). Therefore, we conclude that the effect of galectin-9 targeting therapies may depend on the stage and progression of the disease. Future studies are needed to test the exact functions of galectin-9 under disease conditions, including deep venous thrombosis, cancer-associated thromboembolism, and thromboinflammation.

## 6. Conclusion

Therapeutic strategies disrupting multiple interactions between tumor cells, immune cells, vasculature, and platelets may provide advantages limiting tumor progression and metastasis. However, the biological effects that galectin isoforms are contributed to these processes are variable. Some of the galectin isoforms are strong mediators of inflammation and thrombosis; others are not contributed or not investigated so far. Nevertheless, published results and clinical trials suggest that targeting galectin functions may offer a powerful therapeutic strategy to inhibit not only tumor cell-autonomous attributes but also thrombotic and pro-inflammatory events in the cancer environment. Systematic analysis of tumor stage, platelet and inflammatory markers, and galectin levels in the blood may help to develop therapeutic strategies to inhibit malignancy of tumors, inflammation, and thrombosis.

Studying galectin functions in cancer is surrounded by challenges due to their complex roles in cancer cells and their microenvironment. Some of these challenges are related to the fact that galectin isoforms may have multiple functions in different tumors and depending on the developmental stage and tumor type, also opposite observed functions in tumor progression. Galectins may have pro- or anti-tumor functions. The explanation of these biphasic effects of galectin function is still the subject of many research studies. One interpretation is that the diversity of galectin binding partners or intracellular/extracellular localization of galectins may modify their biological functions during tumor progression. Thus, galectins can act as negative or positive regulators of tumor growth and metastasis in mice and humans.

## Author contributions

LK wrote the manuscript and drafted the figures. AB and EC contributed to the writing. TG critically reviewed the manuscript and contributed to the writing. EM-B conceptualized and wrote the manuscript. All authors contributed to the article and approved the submitted version.

## Funding

This work was supported by the Bayersiches Landesamt für Gesundheit und Lebensmittelsicherheit (BLGL; project number 15–25), Deutsche Forschungsgemeinschaft, CRC TRR152/P15. LK was the recipient of a fellowship from Förderprogramm für Forschung und Lehre (FöFoLe), LMU, Munich, Germany.

## Conflict of interest

The authors declare that the research was conducted in the absence of any commercial or financial relationships that could be construed as a potential conflict of interest.

## Publisher’s note

All claims expressed in this article are solely those of the authors and do not necessarily represent those of their affiliated organizations, or those of the publisher, the editors and the reviewers. Any product that may be evaluated in this article, or claim that may be made by its manufacturer, is not guaranteed or endorsed by the publisher.
